# Macro-scale ore-controlling faults revealed by micro-geochemical anomalies

**DOI:** 10.1038/s41598-019-41019-1

**Published:** 2019-03-13

**Authors:** Emmanuel John M. Carranza, Carlos Roberto de Souza Filho, Paulo Miguel Haddad-Martim, Nagayoshi Katsuta, Ichiko Shimizu

**Affiliations:** 1 Discipline of Geosciences, University of KwaZulu-Natal, Westville Campus, Durban South Africa; 2 Institute of Geosciences, State University of Campinas, Campinas, São Paulo Brazil; 3 Faculty of Education, Gifu University, Gifu, Japan; 4 Department of Earth and Planetary Science, Graduate School of Science, The University of Tokyo, Hongo, Bunkyo-ku, Tokyo Japan

**Keywords:** Geochemistry, Economic geology, Structural geology

## Abstract

Whereas the mechanism of fluid flow, and thus structural control, linked with mineral deposit formation is quite understood, the specific structures that likely provided controls on mineralization at certain geographic scales are not readily known for a given region unless it is well-explored. This contributes uncertainty in mineral prospectivity analysis in poorly-explored regions (or greenfields). Here, because the spatial distribution of mineral deposits has been postulated to be fractals (i.e., the patterns of these features are self-similar across a range of spatial scales), we show for the first time that micro-geochemical anomalies (as proxies of micro-scale patterns of ore minerals), from few discrete parts of the Sossego iron-oxide copper-gold (IOCG) deposit in the Carajás Mineral Province (CMP) of Brazil, exhibit trends of macro-scale faults that are known to have controlled IOCG mineralization in the CMP. The methodology described here, which led to this novel finding, would help towards detecting mineral exploration targets as well as help towards understanding structural controls on mineralization in greenfields.

## Introduction

It has been proposed that the spatial distributions of mineral deposits of specific types are fractals^1–4^, possessing scale-invariance or self-similar properties^5^. Studies on coupled physical processes relevant to mineral formation posit that fluid mixing via chaotic advection is a key ore-forming process that should result in multifractal geometry and spatial distributions of mineral deposits^6–8^. Deforming, chemically reacting systems become critical and produce fractal structures^9^. Thus, despite the complexity of geological processes, including structural controls, linked with mineral deposit formation at different scales, there are systematic patterns in geometry and spatial distributions of mineral deposits.

However, whereas fractal patterns of various geological deformation structures have been studied at various scales^10,11^, fractal spatial distributions of mineral deposits have been studied mainly at regional-scales^1–4^. Some studies have reported that micro-scale fractal geometries of metal-bearing quartz veins in secondary structures are similar to regional-scale fractal geometries of major structures^12,13^, whereas some studies have linked micro-scale fractal distributions of ore/gangue minerals to regional-scale structural processes^14,15^. No studies, however, have linked micro-scale patterns of ore/gangue minerals to local-scale structures at/near mineral deposits to support the proposition that mineral deposits are fractals. This study aimed to explore this knowledge gap, as it could help to understand structural controls on mineral deposit formation across a range of scales and, thus, to map mineral prospectivity to define exploration targets^16^ in poorly-explored frontier regions.

Conceptually, and perhaps ideally, the identified knowledge gap may be addressed by local-scale structural mapping coupled with micro-scale analyses of thin sections of oriented samples of mineralized rocks to determine (a) spatial distributions^17^ of ore minerals and (b) strain^18^ from grains of certain gangue minerals^19,20^. However, the micro-scale analysis of spatial distributions of ore minerals will depend on the choice of ore minerals, which may vary from one location to another and may yield varied results, and the spatial pattern of ore minerals may be obliterated by primary or secondary alteration. Likewise, the micro-scale analysis of strain will depend mainly on the presence of suitable gangue minerals (e.g., quartz, calcite) associated with ore minerals, which may not be present in every mineral deposit. Instead of these micro-scale analyses from ore/gangue minerals, we studied the micro-scale patterns of geochemical anomalies because (a) variations in spatial distributions of elements (termed as geochemical landscapes) in thin sections of oriented samples of mineralized rocks would be a function of ore/gangue mineralogy and (b) it has been proposed that geochemical landscapes are fractals^21^.

Therefore, to show that micro-scale patterns of geochemical anomalies (as proxies of micro-scale patterns of ore/gangue minerals) are linked to local-scale structures at/near mineral deposits in order to support the notion that mineral deposits are fractals, we (a) collected local-scale structural data in the field, (b) collected oriented samples^22^ of mineralized rocks (Extended Data Fig. 1), (c) prepared oriented polished thin sections of oriented rock samples^22^, (d) acquired from oriented polished thin sections images of element concentrations using a scanning X-ray analytical microscope (SXAM)^23^ (see Methods: elemental imaging) because such images have been used for micro-structure analysis^24^ and for analysis of fracture systems as flow paths in granitic rocks from an active orogenic area^25^, (e) subjected the SXAM images of element concentrations to principal component (PC) analysis in order to derive a micro-scale image of geochemical signature (i.e., multi-element association describing the mineralization) (see Methods: principal component analysis), (f) subjected micro-scale PC images of geochemical signature to singularity analysis^26,27^ in order to derive micro-scale images of geochemical anomalies (see Methods: singularity mapping), (g) subjected micro-scale images of geochemical anomalies to spatial neighbourhood analysis to find “geochemical anomaly centres” or “loci of metal enrichment” (see Methods: spatial neighbourhood analysis), (h) subjected “geochemical anomaly centres” per oriented thin section sample to Fry analysis^15^ to describe their trends (see Methods: Fry analysis), and finally (i) compared and contrasted spatial trends of micro-scale geochemical anomalies with those of local-scale faults.

**Figure 1:**
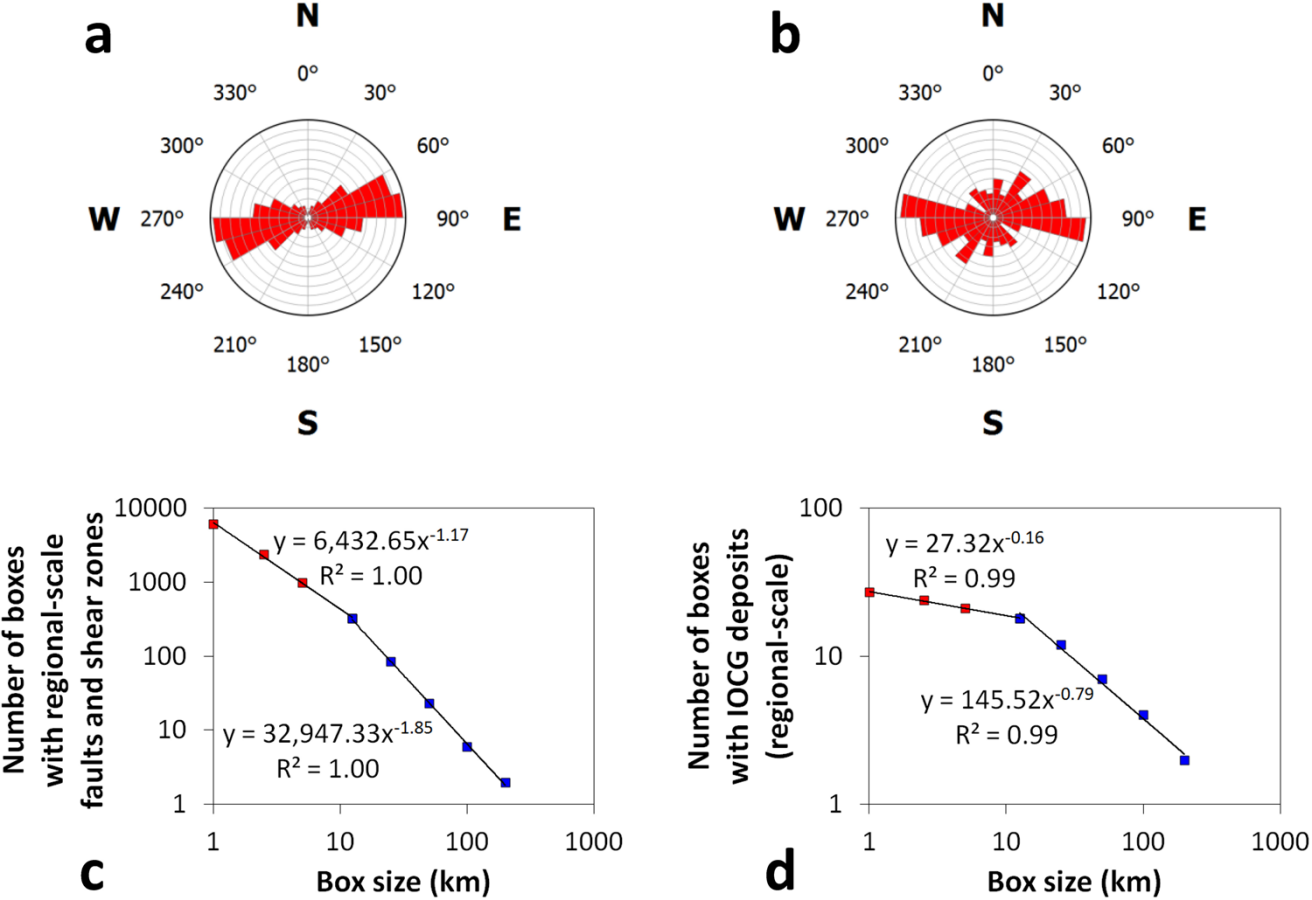
Carajás Mineral Province. Rose diagrams of trends of faults and shear zones (**a**) and trends of Fry plots of IOCG deposits (**b**) are roughly similar. Box-count fractal dimensions of faults and shear zones (**c**) and IOCG deposits (**d**) indicate fractal distributions of these features. See Data Availability for source of data of these features.

We did the research on the Sossego iron oxide-copper-gold (IOCG) deposit in the Carajás Mineral Province (CMP), southeast Amazon Craton, Brazil, for which we recently published new interpretations of regional-scale structural controls on IOCG mineralization based on analyses of regional-scale trends of IOCG deposits^28^. We chose to study a mineral deposit in the CMP because it contains several world-class ore deposits and because it contains the largest known concentration of large-tonnage (>100 million tons) IOCG deposits^29^. We chose to study an IOCG deposit because it is a class of mineral deposits known for its ubiquitous structural control^30^. Regional-scale faults and shear zones in the CMP mostly trend nearly E–W (Fig. 1a), which is adopted by the IOCG deposits (Fig. 1b). However, apparently the IOCG deposits were hosted more favourably by structures with the less frequent trends of N70°W rather than by structures with the most frequent trends of N70°E. In addition, regional-scale faults and shear zones in the CMP exhibit fractal distribution (Fig. 1c), which is adopted by the IOCG deposits as well (Fig. 1d).

**Figure 2:**
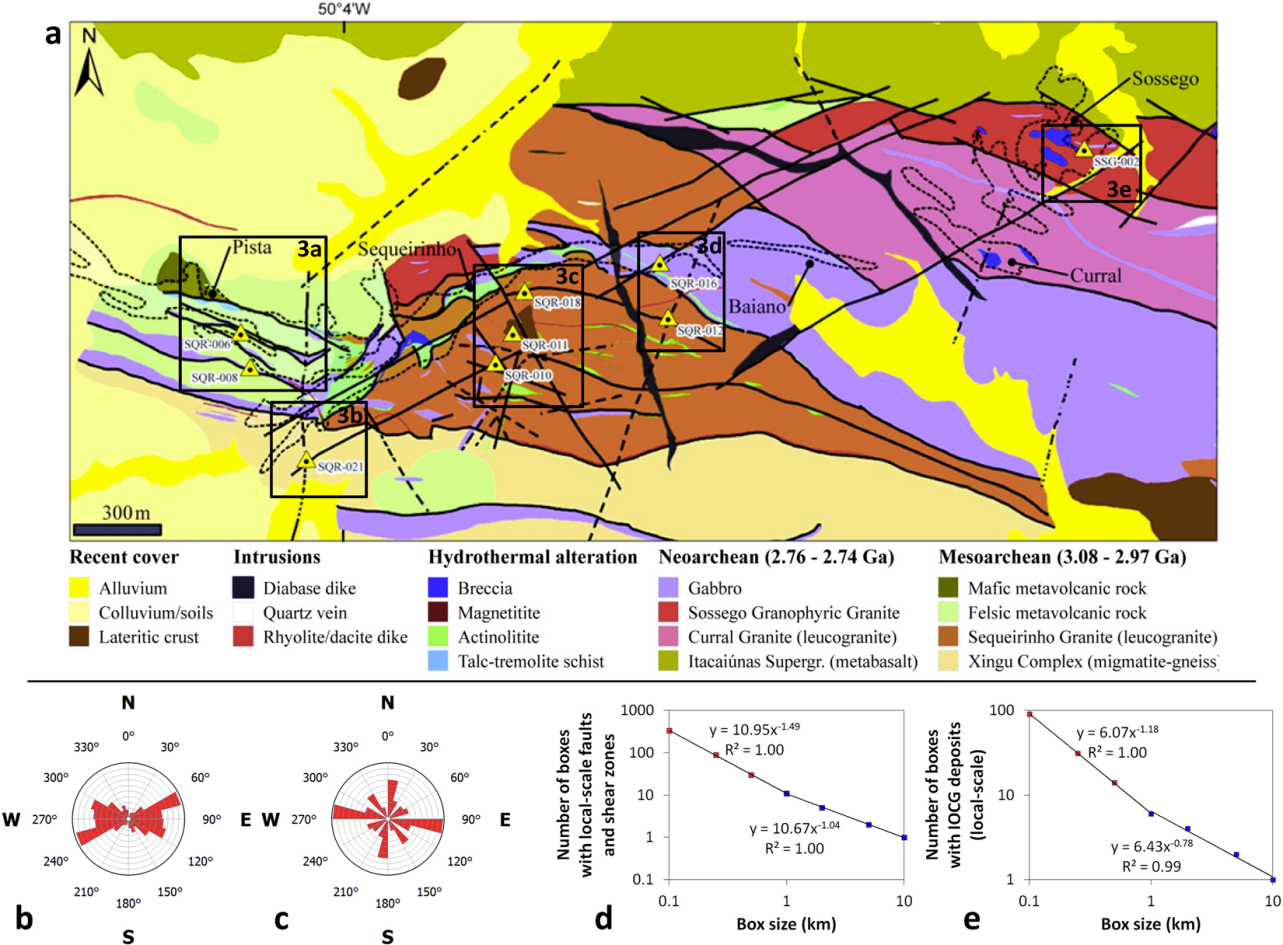
IOCG mineralization at Sossego. (**a**) Simplified geological map (slightly modified from unpublished map of VALE Company). Transparent polygons with dotted outlines are pre-2008 surface projections of ore bodies delineated by VALE company^31^. Labelled triangles with black dots are locations of oriented samples of mineralized rocks. Labelled rectangles are parts of the area with results for the oriented samples used in the study (Fig. 3). Rose diagrams of trends of faults and shear zones (**b**) and trends of Fry plots IOCG deposits (**c**) are roughly similar. Box-count fractal dimensions of faults and shear zones (**d**) and IOCG deposits (**e**) indicate fractal distributions of these features. See Data Availability for source of data of these features.

Sossego, located ~30 km NNW of Rio de Janeiro, is a world-class IOCG deposit being mined by VALE Inc. since 2004, among the largest IOCG deposits in the CMP. It contains ~245 Mt of ore at 1.1 wt. % Cu and 0.28 g/t Au^29^. It is situated near the contact between gneisses/migmatites of the ~2.8 Ga Xingu Complex and metabasalts of the ~2.76 Ga Itacaiúnas Supergroup, on the northern limits of the Canaã shear zone, a set of sub-vertical, WNW-ESE-trending ductile shear zones^31^. Mineralization at Sossego is distributed among five ore bodies, which form two groups according to their positions and characteristics: Pista–Sequeirinho–Baiano and Sossego–Curral (Fig. 2a). Local-scale faults and shear zones at Sossego mostly trend ENE–WSW and WNW–ESE (Fig. 2a,b) and exhibit fractal distribution (Fig. 2d), and the Sossego deposit has adopted a main WNW–ESE trend (Fig. 2c) as well as a fractal distribution (Fig. 2e). However, there are other local-scale trends in IOCG mineralization that do not reflect the main local-scale trends faults and shear zones, e.g., the nearly N–S trend (Fig. 2c), which is related to nearly N–S-trending structures as at sample SQR-021(Fig. 2a).

The similarity of local-scale trends of faults and shear zones at Sossego to the regional-scale trends of such structures in the CMP as well as the similarity of local-scale trends of IOCG mineralization to regional-scale trends of IOCG deposits clearly show that either set of objects is a fractal (i.e., the parts of a pattern look similar to the whole pattern). The similarity of local-scale trends of IOCG mineralization (Fig. 2c) to regional-scale trends of faults and shear zones (Fig. 1b), which is consistent with studies on IOCG and other types of mineralization elsewhere^32–36^, reflects structural control by regional-scale structures on mineralization at local scales.

The similarity of micro-scale trends of geochemical anomalies (as proxies of micro-scale patterns of ore/gangue minerals) to local-scale trends of structures at/near mineral deposits is illustrated from nine thin sections of oriented samples of mineralized rocks that we collected from different parts of the Sossego deposit along its longitudinal and transverse axes (Fig. 2a).

## Results

**Figure 3:**
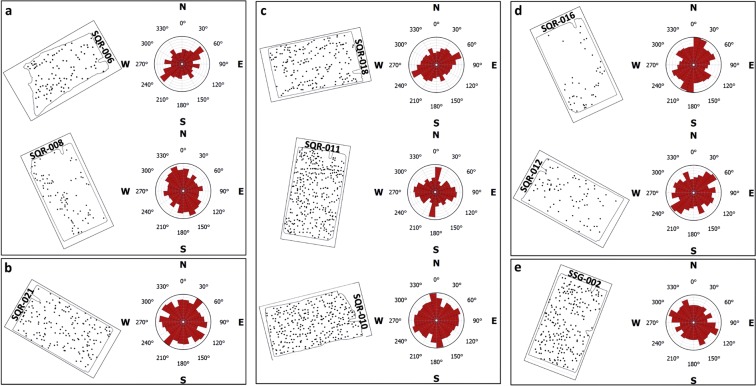
Trends of micro-scale geochemical anomalies in the Sossego deposit. The thin section stamps are shown in their correct orientations with respect to the geological map (see Fig. 2). Details of micro-scale geochemical anomaly centres (black dots) in thin sections of oriented samples of mineralized rocks are shown in Extended Data Figure 15, and details of their corresponding rose diagrams of Fry plots of micro-anomaly centres are shown Extended Data Figures 17–25. Results shown for samples: **(a**) SQR-006 and SQR-008; (**b**) SQR-21; (**c**) SQR-010, SQR-011 and SQR-018; (**d**) SQR-012 and SQR-016; and (**e**) SSQ-002.

Micro-scale geochemical anomalies in sample SQR-006 (Fig. 3a) show: (a) a major NE–SW trend, which is likely linked to the south-westward extension of a NE–SW-trending shear zone located ~200 m NE of this sample (Fig. 2a); and (b) minor NW–SE and WNW–ESE trends, which reflect control by nearby faults with the same trends.

Micro-scale geochemical anomalies in sample SQR-008 (Fig. 3a) show: (a) major NNW–SSE to NW–SE trends, which are likely linked to the NNW–SSE- to NW–SE-trending faults located ~50 to ~100 m north of this sample (Fig. 2a); (b) a major N–S trend, which suggests the presence of N–S-trending shear zone at this sample location similar to the one located ~150 m to the east; (c) minor E–W trend, reflecting control by the nearly E–W-trending fault at the locations of this sample; and (d) minor NE–SW trend, which suggests the presence of NE–SW-trending shear zone at this sample location similar to the one located ~300 m to the northeast.

Micro-geochemical anomalies in sample SQR-021 (Fig. 3b) show: (a) a major NE–SW trend, reflecting control by a major NE–SW-trending fault at this sample location (Fig. 2a); (b) secondary N–S trends, reflecting control by a NE–SW-trending shear zones at this sample location; and (c) secondary NW–SE- to WNW–ESE trends, which suggest the presence of structures with the same trends at/around this sample location.

Micro-scale geochemical anomalies in sample SQR-010 (Fig. 3c) show: (a) major NNW–SSE to secondary NNE–SSW trends, which can be linked to NNE–SSW-trending shear zones south and east of this sample location (Fig. 2a); and (b) secondary ENE–WSW trends, which can be linked to faults with the same trend at this sample location.

Micro-geochemical anomalies in sample SQR-011 (Fig. 3c) show: (a) a major NNE–SSW trend, which can be linked to shear zones with the same trend at this sample location (Fig. 2a); and (b) secondary WNW–ESE to ENE–WSW trends, which reflect control by WNW–ESE-trending shear zones similar to the one located south of this sample point.

Micro-scale geochemical anomalies at sample SQR-018 (Fig. 3c) show: (a) a major ENE–WSW trend, which reflects control by faults with the same trend located at and SW of this sample location (Fig. 2a); (b) a secondary NNW–SSE trend, which suggests control by faults of the same structure at this sample location.

Micro-scale geochemical anomalies in sample SQR-012 (Fig. 3d) show: (a) a major NE–SW trend, which is likely linked to a NNE–SSW-trending shear zone or suggesting the presence of unmapped NW–SE-trending structures at this sample location (Fig. 2a); (b) secondary WNW–ESE trend, which reflects control by a major fault with the same trend at this sample location; and (c) secondary NNW–SSE and ENE–WSW trends, which suggest the presence of structures with these trends at/near this sample location.

Micro-scale geochemical anomalies in sample SQR-016 (Fig. 3d) show: (a) a major NNE–SSW trend, which reflects control by a shear zone with the same trend at this sample location like the one present ~120 m east of it (Fig. 2a); (b) secondary NW–SE trends, which suggest the presence at this sample location of faults with the same trends like the one present ~50 m south of it; and (c) secondary ENE–WSW to NE–SW trends, which reflect control by a fault with the same trend at this sample location.

Micro-scale geochemical anomalies in sample SSG-002 (Fig. 3e) show: (a) major WNW–ESE to NW–SE trends, which suggest the presence of and reflect control by faults with the same trends like those ~150 m southwest and ~100 m northeast of this sample location (Fig. 2a); and (b) secondary NE – SW trends, which suggest the presence of and reflect control by faults with the same trends like the one ~100 m southeast of this sample location.

## Discussion

The above observations clearly illustrate for the first time that trends of micro-geochemical anomalies of IOCGs are similar to (a) trends of local-scale faults and shear zones, supporting the knowledge of structural control on IOCG mineralization at Sossego^28,31,37^, and (b) local- to regional-scale trends of IOCG mineralization (Table 1), supporting the proposition that the spatial distributions of mineral deposits of specific types are fractals^1–4^. The trends of micro-geochemical anomalies that we report here are strongly consistent with the trends of chalcopyrite grains that we have measured in thin sections^38^ and with the trends of regional-, district- and local-scale faults and shear zones that are currently considered for publication in another journal. In this forthcoming paper, our analyses suggest that the initial phase of hydrothermal alteration at Sossego was coeval with the generation of several local-scale dilational jogs. In addition, our earlier regional-scale Fry analysis of the CMP IOCG deposits, including Sossego, reveals a sigmoidal pattern^28^ that is consistent with the Carajás sigmoid that was interpreted^39^ to have formed during 2.7–2.6 Ga under dextral transtension by the subsidence of supracrustal units into dilatational jogs. These novel findings are critical to geological mapping, understanding of mineralization controls and mineral exploration in frontier regions (or so-called greenfields) where the geology is poorly-mapped. In such regions, the collection of oriented samples at outcrops of mineral showings and the application of the methodology described here can result in the recognition of trends of unmapped faults and, thus, can guide detection and mapping of geological structures, say by remote sensing using satellite imagery and/or airborne geophysical data. In greenfields where structural control on mineralization is poorly-understood, the application of the methodology presented here can result in the recognition of trends of structures that are probably macro-scale controls of mineralization, which, in turn, can aid in predictive mapping of exploration targets^16,40,41^.

**Table 1:** Trends of micro-scale geochemical anomalies in the Sossego deposit and their corresponding trends in local- to regional-scale spatial distribution of IOCG mineralization at Sossego and the CMP, respectively.

Samples	Trends in micro-scale geochemical anomalies	Corresponding trends in local- to regional-scale spatial distribution of IOCG mineralization
SQR-006 (Fig. 3a)	(a) major NE–SW trend; and	(a) minor regional-scale NE–SW trend of IOCG deposits (Fig. 1c);
	(b) minor NW–SE and WNW–ESE trends	(b) major regional and local-scale WNW–ESE trends of IOCG mineralization (Figs 1c, 2c)
SQR-008 (Fig. 3a)	(a) major NNW–SSE to NW–SE trends;	(a) minor local-scale NW–SE trend mineralization (Fig. 2c).
	(b) major N–S trend;	(b) minor local-scale N–S trend of IOCG mineralization (Fig. 2c);
	(c) minor E–W trend; and	(c) major regional- and local-scale WNW–ESE trends of IOCG mineralization (Figs 1c, 2c); and
	(d) minor NE–SW trend	(d) minor regional-scale NE–SW trend of IOCG deposits (Fig. 1c).
SQR-021 (Fig. 3b)	(a) major NE–SW trend;	(a) minor regional-scale NE–SW trend of IOCG deposits (Fig. 1c);
	(b) secondary N–S trends; and	(b) secondary local-scale NNE–SSW trend in IOCG mineralization (Fig. 2c); and
	(c) secondary NW–SE- to WNW–ESE trends	(c) major regional- and local-scale WNW–ESE trends of IOCG mineralization (Figs 1c, 2c)
SQR-010 (Fig. 3c)	(a) major NNW–SSE to secondary NNE–SSW trends;	(a) minor local-scale NNE–SSW trend of IOCG mineralization (Fig. 2c); and
	(b) secondary ENE–WSW trends	(b) minor regional-scale NE–SW trend of IOCG deposits (Fig. 1c)
SQR-011 (Fig. 3c)	(a) major NNE–SSW trend; and	(a) minor local-scale NNE–SSW trend of IOCG mineralization (Fig. 2c); and
	(b) secondary WNW –ESE to ENE–WSW trends	(b) major regional- to local-scale WNW –ESE trends of IOCG mineralization (Figs 1c, 2c)
SQR-018 (Fig. 3c)	(a) major ENE–WSW trend; and	(a) secondary regional-scale ENE–WSW trend of IOCG deposits (Fig. 1c); and
	(b) secondary NNW–SSE trend	(b) minor local-scale NW–SE trend of IOCG mineralization (Fig. 2c)
SQR-012 (Fig. 3d)	(a) major NW–SE trend;	(a) minor local-scale NW–SE trend of IOCG mineralization (Fig. 2c);
	(b) secondary WNW–ESE and ENE–WSW trends; and	(b) major regional- to local-scale WNW–ESE trends of IOCG mineralization (Figs 1c, 2c); and
	(c) secondary NNW–SSE trend	(c) minor local-scale NW – SE trend in IOCG mineralization (Fig. 2c)
SQR-016 (Fig. 3d)	(a) major NNE–SSW trend;	(a) secondary local-scale NNE–SSW trend in IOCG mineralization (Fig. 2c);
	(b) secondary NW–SE trends; and	(b) minor local-scale NW–SE trends of IOCG mineralization (Fig. 2c); and
	(c) secondary ENE–WSW to NE–SW trends	(c) minor regional-scale NE–SW trend of IOCG deposits (Fig. 1c)
SSG-002 (Fig. 3e)	(a) major WNW–ESE to NW–SE trends; and	(a) major regional- to local-scale WNW–ESE trends of IOCG mineralization (Figs 1c, 2c); and
	(b) secondary NE–SW trends	(b) minor regional-scale NE–SW trend of IOCG deposits (Fig. 1c)

The stimulus behind the methodology discussed here is the synthesis of ideas regarding (a) fluid flow in porous media as a factor of mineralization^42,43^, (b) imaging microscopy of porous media to model micro-scale fluid flow^44,45^, (c) singularity as a fractal property of anomalous amount of energy release or mass (i.e., metal/element) accumulation during the formation of a mineral deposit^26,27^, and (d) point pattern analysis^15^ of micro-scale ‘centres of geochemical anomalies’ (i.e., singularities as proxies of loci of multi-element accumulation at/along pores in rocks). This paper has shown the usefulness of SXAM images^24^ for the purpose of this study. This study underscores the need for the orientation of rock samples from outcrops in the field^22^ and the orientation of drill cores as standard rock sampling protocols in geology. This research proves graphically the fractal nature of mineral deposits, which has important implications not only for economic geology but for the geosciences in general.

## Methods

### Elemental imaging

From oriented samples of mineralized rocks (Extended Data Fig. 1), we prepared polished thin sections (Extended Data Fig. 2) from which we acquired images of element concentrations using the SXAM at Gifu University in Japan.

The SXAM system that we used (model XGT-2000V by Horiba Scientific) is an X-ray fluorescence analyser that uses a continuous high energy X-ray beam (Rh anode 50 kV 1 mA), 100 μm in diameter, focused with a guide tube and irradiated perpendicular to the surface of a sample^23,24^. X-ray fluorescence from the surface of sample is observed by a high-purity Si detector of an energy-dispersive spectrometer. In this SXAM system, a sample is mounted on a PC-controllable motor-driven X–Y stage placed in an open space outside the vacuum chamber. Therefore, among the elements that SXAM can analyze^46^ (i.e., sodium to uranium), it is difficult to obtain compositional images for the lightest elements (e.g., Al), especially if these are present in very low concentrations, because low-energy fluorescence X-rays are absorbed by air and the film of the window. However, compared to other elemental imaging methods like electron probe micro-analyser or scanning electron microscopy with energy dispersive X-ray analyser, the SXAM can measure two-dimensional distribution of chemical elements over a significantly wider range of scales (e.g., it can acquire elemental images for a sample set on a standard size 24 × 46 mm petrographic thin section) because the scanning area can be set from 2.56 × 2.56 mm with a resolution of 10 mm, to roughly 200 × 400 mm with a resolution of 0.78 mm. The count number of fluorescence X-rays of each element is stored as a digital image that consists of pixels; each pixel corresponding to a position on the sample surface.

We analysed our polished thin sections of oriented samples of mineralized rocks for eight elements (Al, Si, S, K, Ca, Ti, Fe and Cu) (Extended Data Figs 3–11), which are representative of the suite of ore/gangue minerals^31,47^ in these rock samples (i.e., chalcopyrite (CuFeS_2_) and pyrite (FeS_2_) representing ore minerals; biotite (K(Mg,Fe)_3_(AlSi_3_O_10_)(F,OH)_2_), magnetite (Fe_3_O_4_), and quartz (SiO_2_) representing gangue minerals that comprise potassic alteration in the Sossego–Curral ore bodies; albite (NaAlSi_3_O_8_), actinolite (Ca_2_(Mg,Fe)_5_Si_8_O_22_(OH)_2_), magnetite, titanite (CaTiSiO_5_), epidote (Ca_2_(Al,Fe)_2_(SiO_4_)_3_(OH)), and calcite (CaCO_3_) representing gangue minerals that typically comprise sodic and sodic-calcic alteration in the Pista–Sequeirinho–Baiano ore bodies). It should be noted that chalcopyrite is the main ore mineral in the Sossego IOCG deposit^31,47^, and so it is also in most of our samples (see Extended Data Fig. 12).

### Principal component analysis

To derive from each set of SXAM images of element concentrations a micro-scale image of geochemical signature (i.e., multi-element association) depicting the mineralization, we followed the traditional approach of applying principal component (PC) analysis to maps/images of concentrations of multiple elements to derive maps/images of multi-element associations (i.e., geochemical signatures) depicting geological processes of interest^48,49^. Nowadays this approach makes use of logratio-transformed geochemical data^50,51^ because element compositions that are measured/expressed as ratios (i.e., as %, ppm, ppb, etc.) are constrained to a constant sum and, thus, represent closed number systems, which render spurious results in multivariate (e.g., PC) analysis^52^. However, logratio transformation of element concentrations in the SXAM images is not necessary because these data (i.e., X-ray counts) do not represent closed number systems.

Each set of SXAM images of element concentration per thin section sample (Extended Data Figs 3–11) is subjected to PC analysis, yielding a number of PCs equal to the number of input images. The PC depicting the geochemical signature of mineralization is interpreted according to the signs and magnitudes of the loadings (eigenvectors) of each element on a PC. For each pixel in an image, a PC score can be calculated as the linear sum of products between element loading and element concentration. We performed the PC analysis in the GIS (geographic information system) software ILWIS, to which we imported the digital data of the SXAM images saved as ASCII (or tab delimited text) files.

The geochemical signature of mineralization in sample SQR-006 is represented by PC1, which depicts a Fe-Ti association that explains about 64% of the multivariate data variance (Extended Data Table 1). The high negative loading on Fe in PC1 reflects pyrite (FeS_2_) whereas the low negative loading on Ti reflects titanite (CaTiSiO_5_) representing calcic alteration. Because of the negative loading on Fe in PC1, the PC1 scores are negated (i.e., multiplied by −1) so that low to high negated PC1 scores reflect increasing intensity of mineralization or metal enrichment. The spatial distribution of negated PC1 scores depicting the Fe-Ti association is shown in Extended Data Fig. 13a.

The geochemical signature of mineralization in sample SQR-008 is represented by PC2, which depicts an antipathetic association between Cu and Ca-Ti and explains about 7% of the multivariate data variance (Extended Data Table 2). The high negative loading on Cu in PC2 reflects chalcopyrite (CuFeS_2_) whereas the intermediate and low positive loadings on Ca and Ti, respectively, reflect a suite of minerals (e.g., epidote (Ca_2_(Al,Fe)_2_(SiO4)_3_(OH)) and titanite (CaTiSiO_5_)) that comprise calcic alteration. Because of the negative loading on Cu in PC2, the PC2 scores are negated (i.e., multiplied by −1) so that low to high negated PC2 scores reflect increasing intensity of mineralization or metal enrichment. The spatial distribution of negated PC2 scores, which represent Cu mineralization, is shown in Extended Data Fig. 13b.

The geochemical signature of mineralization in sample SQR-010 is represented by PC1, which depicts mainly Cu and explains about 98% of the multivariate data variance (Extended Data Table 3). The high positive loading on Cu in PC1 reflects chalcopyrite (CuFeS_2_). The spatial distribution of PC1 scores, which represent Cu mineralization, is shown in Extended Data Fig. 13c.

The geochemical signature of mineralization in sample SQR-011 is represented by PC1, which depicts a Fe-Cu association that is antipathetic with Ca and explains about 77% of the multivariate data variance (Extended Data Table 4). The high and low positive loading on Fe and Cu, respectively, in PC1 reflects pyrite (FeS_2_) and chalcopyrite (CuFeS_2_) whereas the intermediate negative loading on Ca reflects a suite of mineral representing calcic alteration. The spatial distribution of PC1 scores, which represents Fe-Cu mineralization, is shown in Extended Data Fig. 13d.

The geochemical signature of mineralization in sample SQR-012 is represented by PC1, which depicts mainly Cu and explains about 89% of the multivariate data variance (Extended Data Table 5). The high positive loading on Cu in PC1 reflects chalcopyrite (CuFeS_2_). The spatial distribution of PC1, which represents Cu mineralization, in the thin section sample is shown in Extended Data Fig. 13e.

The geochemical signature of mineralization in sample SQR-016 is represented by PC4, which depicts Cu-Ti association and explains roughly 1% of the multivariate data variance (Extended Data Table 6). The high and intermediate negative loadings on Cu and Ti in PC1 reflect chalcopyrite (CuFeS_2_) and titanite (CaTiSiO_5_), respectively, the latter representing calcic alteration. The PC1, with high positive loading on Fe and explains about 96% of the multivariate data variance, mainly represents hematite (Fe_2_O_3_) alteration and was not considered as the geochemical signature of mineralization. Because of the negative loading on Cu in PC4, the PC4 scores are negated (i.e., multiplied by −1) so that low to high negated PC4 scores reflect increasing intensity of mineralization or metal enrichment. The spatial distribution of PC4 scores, which represent Cu mineralization, is shown in Extended Data Fig. 13f.

The geochemical signature of mineralization in sample SQR-018 is represented by PC2, which depicts Cu-K association and explains roughly 5% of the multivariate data variance (Extended Data Table 7). The high and low negative loadings on Cu and K in PC2 reflect chalcopyrite (CuFeS_2_) and biotite (K(Mg,Fe)_3_(AlSi_3_O_10_)(F,OH)_2_), respectively, the latter representing potassic alteration. The PC1, with high positive loading on Fe and explains about 94% of the multivariate data variance, mainly represents hematite (Fe_2_O_3_) alteration and was not considered as the geochemical signature of mineralization. Because of the negative loading on Cu in PC2, the PC2 scores are negated (i.e., multiplied by −1) so that low to high negated PC2 scores reflect increasing intensity of mineralization or metal enrichment. The spatial distribution of PC2 scores, which represent Cu mineralization, is shown in Extended Data Fig. 13g.

The geochemical signature of mineralization in sample SQR-021 is represented by PC2, which depicts an antipathetic association between Cu and Fe and explains about 15% of the multivariate data variance (Extended Data Table 8). The high negative loading on Cu in PC2 reflects chalcopyrite (CuFeS_2_) whereas the low positive loading on Fe represents hematite (Fe_2_O_3_). Because of the negative loading on Cu in PC2, the PC2 scores are negated (i.e., multiplied by −1) so that low to high negated PC2 scores reflect increasing intensity of mineralization or metal enrichment. The spatial distribution of PC2 scores, which represent Cu mineralization, is shown in Extended Data Fig. 13h.

The geochemical signature of mineralization in sample SSG-002 is represented by PC1, which depicts an antipathetic association between Fe-Cu and Ca and explains about 70% of the multivariate data variance (Extended Data Table 9). The high and intermediate positive loadings on Fe and Cu in PC1 reflect pyrite (FeS_2_) and chalcopyrite (CuFeS_2_), respectively, whereas the intermediate negative loading on Ca represents a suite of minerals comprising calcic alteration. The spatial distribution of PC1 scores, which represent Fe-Cu mineralization, is shown in Extended Data Fig. 13i.

### Singularity mapping

We subjected each of the micro-scale PC images of geochemical signature of mineralization (Extended Data Fig. 13) to singularity analysis^26,27^ in order to derive micro-scale images of geochemical anomalies. Singularity is defined, from a geoscience viewpoint, as a special geological event associated with anomalous energy release or material accumulation that take place during restricted spatial–temporal intervals^26^. The method for mapping singularity indices (denoted as *α*) has been developed and described by Cheng^26,27^ and is not repeated here. However, we used a MATLAB-based software that is available from its developers^53^. To run the singularity mapping algorithm in this software, image data in raster (i.e., pixel-based) format must be converted to ASCII (or tab delimited text file) format. The output file of singularity indices is also in ASCII format, which can be imported to software for image analysis (and in this study we used the ILWIS GIS software).

The images of singularity indices are shown in Extended Data Fig. 14. In each of these images, pixels with *α* < 2 are positions where positive singularity exists (i.e., where element/metal enrichment increased as area decreased), pixels with *α* > 2 are positions where negative singularity exists (i.e., where element/metal enrichment decreased as area decreased), and pixels with *α* = 2 are positions where neither positive nor negative singularity did not occur^26^. Therefore, geochemical anomalies are defined by pixels with *α* < 2 (Extended Data Fig. 15).

### Spatial neighbourhood analysis

We subjected each of the micro-scale images of geochemical anomalies (Extended Data Fig. 15) to spatial neighbourhood analysis to locate positions that are likely “geochemical anomaly centres” or “loci of metal enrichment”. In a neighbourhood of pixels in an image of singularity indices, the “geochemical anomaly centre” or “locus of metal enrichment” is a pixel with the lowest singularity index because geochemical anomalies are defined by pixels with *α* < 2. To locate such pixels (denoted as “pits”), we ran a spatial neighbourhood algorithm, namely Pit = NBMINP(MAP#) = 5, where MAP# is a neighbourhood matrix (i.e., a 3 × 3 kernel filter) will move over input map an image (each of the SXAM images) and position (denoted by #) found by the matrix will be ‘retrieved’ according to the command NBMINP, which returns the position of the neighbour that has the smallest value. This algorithm is commonly used to identify local pits in a digital elevation model. In a 3 × 3 kernel filter, the pixels are coded with numbers 1 to 9 from left to right and from top to bottom; thus, the central pixel is coded 5. If the “Pit” algorithm finds multiple neighbour pixels having the same smallest value, then the precedence for retrieving the “pit” is: central pixel number 5, and then pixels 1, 2, 3, 4, 6, 7, 8, 9. The positions of “geochemical anomaly centres” or “loci of metal enrichment” in individual thin section specimen are shown in Extended Data Fig. 16.

### Fry analysis

To describe trends of “geochemical anomaly centres” per oriented thin section sample, we used Fry analysis^15^. This is a graphical method of spatial autocorrelation analysis of objects depicted as points, whereby each and every point is used as origin for translation. The method plots translations (so-called Fry plots) of point objects by using each and every point as a centre or origin for translation. Detailed description, with illustration, for creating Fry plots can be found in Carranza^16^ and is not repeated here. Fry plots have been developed, in the late 1970s, originally for the investigation of strain and strain partitioning in rocks^15,54^. Since two decades later, Fry plots have been used to analyse spatial distributions of mineral deposits^16,28,55–58^ and geothermal fields^59^ in order to infer their structural controls.

To visualize trends in Fry plots a rose diagram can be created for (a) all pairs of translated points and (b) pairs of translated point within a specified distance from each other. The former case may reveal trends due to processes operating at macro-scales but may also show a trend that is an artefact of the shape of the study area (e.g., a major trend due to the longer axis of a thin section), whereas the latter case may reveal trends due to processes operating at micro-scales. For the latter case, it is instructive to use a distance within which there is maximum probability of only two neighbouring points (i.e., analysis of trends between any two neighbouring “geochemical anomaly centres”). This distance can be determined via point pattern analysis^60^.

The Fry plots of “geochemical anomaly centres” and corresponding rose diagrams of trends between pairs of Fry points at specified distances in the thin sections of the studied samples are shown in Extended Data Figs 17–25. Pairs of Fry points at specified distances in thin sections of samples SQR-006, SQR-008, SQR-010 SQR-011, SQR-016, and SQR-021 show major trends that follow the trends of the thin sections’ long axes. However, these major trends are likely real because pairs of Fry points at specified distances in thin sections of samples SQR-012, SQR-018, SQR-021, and SSG-002 show major trends that do not follow the trends of the thin sections’ long axes.

## Supplementary information

Extended data figures and tables

## Data Availability

For trends of regional-scale faults and shear zones in the CMP (Fig. 1), we obtained the data from regional lithological-structural map of the CMP (Vasquez, M.L. & Rosa-Costa, L.T. Geologia e Recursos Minerais do Estado do Pará: Sistema de Informações Geográficas - SIG: texto explicativo dos mapas Geológico e Tectônico e de Recursos Minerais do Estado do Pará. CPRM (Companhia de Pesquisa de Recursos Minerais), Belém (328 pp) (2008). For trends of local-scale faults and shear zones at Sossego, we obtained data from the geological map of Sossego (Fig. 2) that is available in Monteiro *et al*.^31^. For trends of micro-scale geochemical anomalies, we collected oriented samples of rocks from which we obtained micro-scale images of geochemical anomalies (Extended Data Figs 1–11 and 13–16). The digital data of the SXAM images, from which we obtained micro-scale images of geochemical anomalies, are available as ASCII (or tab delimited text) files at https://osf.io/5cvhz/.

## Code Availability

We used the publicly available GIS software in this study: ILWIS 3.3 (http://www.itc.nl/ilwis/downloads/ilwis33.asp). We also used a MATLAB-based software for fractal/multifractal modeling, which is available from its developers^53^.
