# Author Correction: Macro-scale ore-controlling faults revealed by micro-geochemical anomalies

**DOI:** 10.1038/s41598-019-44433-7

**Published:** 2019-05-28

**Authors:** Emmanuel John M. Carranza, Carlos Roberto de Souza Filho, Paulo Miguel Haddad-Martim, Nagayoshi Katsuta, Ichiko Shimizu

**Affiliations:** 1 Discipline of Geosciences, University of KwaZulu-Natal, Westville Campus, Durban, South Africa; 2 Institute of Geosciences, State University of Campinas, Campinas, São Paulo Brazil; 3 Faculty of Education, Gifu University, Gifu, Japan; 4 Department of Earth and Planetary Science, Graduate School of Science, The University of Tokyo, Hongo, Bunkyo-ku, Tokyo Japan

Correction to: *Scientific Reports* 10.1038/s41598-019-41019-1, published online 13 March 2019

The original version of this Article contained an error in the spelling of the author Nagayoshi Katsuta, which was incorrectly given as Katsuta Nagayoshi. In addition, the Article contained an error in Affiliation 3, which was incorrectly given as ‘Department of Education, Gifu University, Gifu, Japan’. The correct affiliation is listed below:

Faculty of Education, Gifu University, Gifu, Japan

These errors have now been corrected in the PDF and HTML versions of the Article, and in the accompanying Supplementary Information file.

