# Supplementary material for: Macro-scale ore-controlling faults revealed by micro-geochemical anomalies
Source: Sci Rep. 2019 Mar 13;9:4410. doi: 10.1038/s41598-019-41019-1 (PMC6416276; doi:10.1038/s41598-019-41019-1)
Supplement: Supplementary file 1 — Extended data figures and tables [file 41598_2019_41019_MOESM1_ESM.pdf]

Macro-scale ore-controlling faults revealed by micro-geochemical anomalies

Emmanuel John M. Carranza[1,*], Carlos Roberto de Souza Filho[2], Paulo Miguel Haddad-Martim[2], Nagayoshi Katsuta[3], Ichiko Shimizu[4]

Supplementary information

Extended data figures. Extended data tables.

[1]Discipline of Geosciences, University of KwaZulu-Natal, Westville, Campus, Durban, South Africa. [2]Institute of Geosciences, State University of Campinas, Campinas, São Paulo, Brazil. [3]Faculty of Education, Gifu University, Gifu, Japan. [4]Department of Earth and Planetary Science, Graduate School of Science, The University of Tokyo, Hongo, Bunkyo-ku, Tokyo, Japan. *Correspondence to ejmcarranza@gmail.com

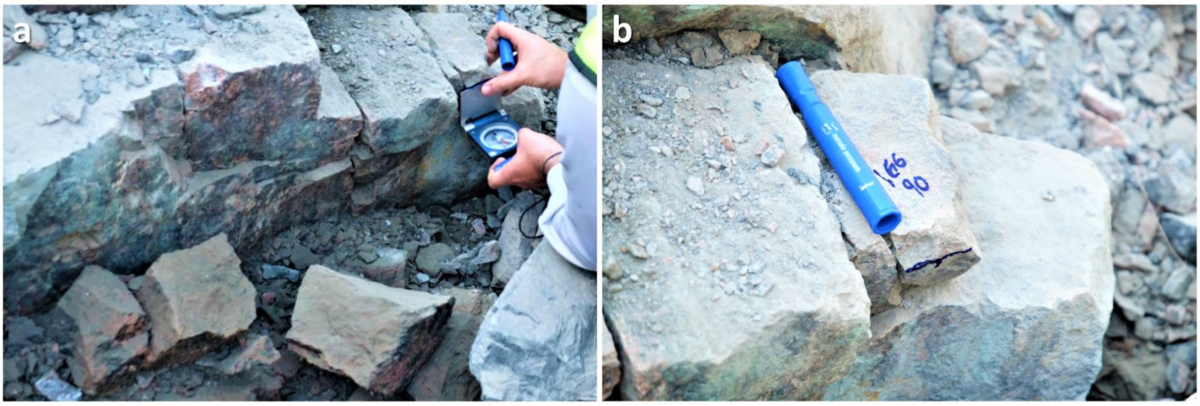

Extended Data Figure 1 | Collection of oriented rock samples. (**a**) An exposed reference plane of a geological structure (e.g., fault, fracture, joint, etc.) in the mineralized rock is chosen and measured for its strike (orientation) and dip (inclination) using a compass/clinometer. (**b**) Strike (first number) and dip (second number) are labelled on a surface of the rock to be sampled, and a strike/dip symbol is marked on the reference plane that was measured. Marking pen is shown for scale. The labelled/marked part of the mineralized rock is then extracted/sampled with aid of a geological hammer.

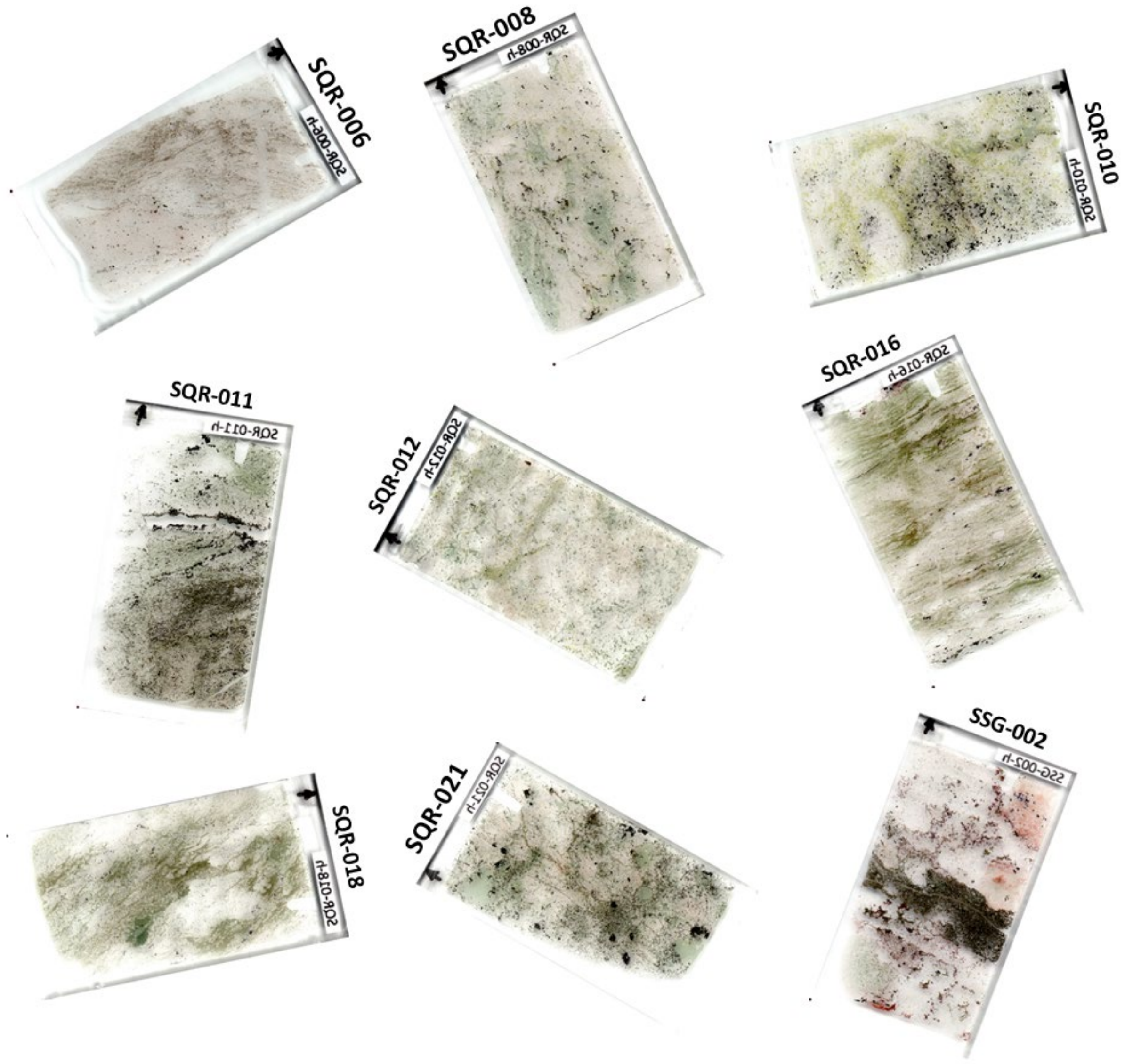

Extended Data Figure 2 | Polished thin sections of oriented rock samples. The oriented rock samples were cut perpendicular to the vertical, and so the nine thin sections used in this study are shown in their correct orientations with respect to the geological map (i.e., the North-South orientation is parallel to the long-side of this page).

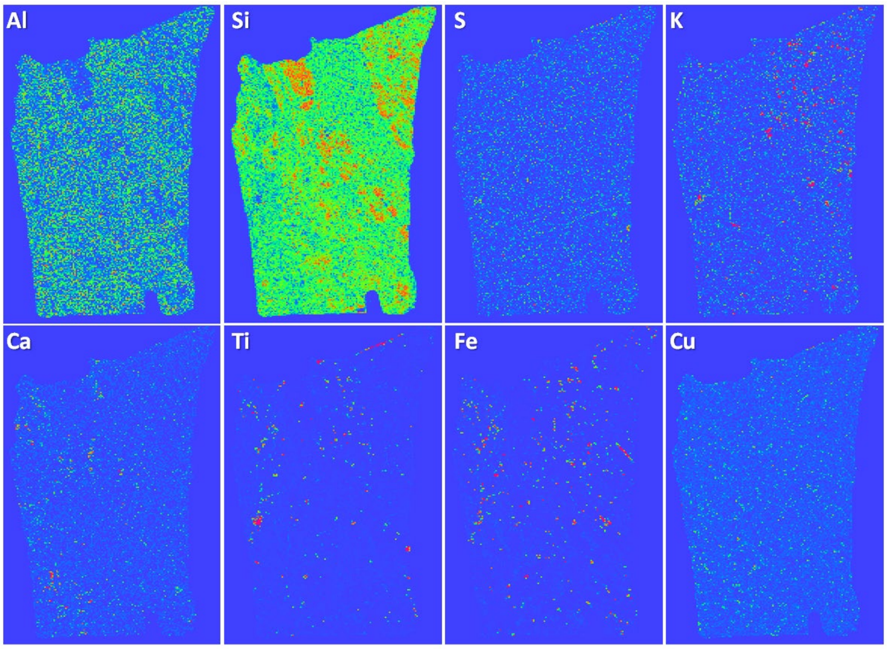

SQR-006

Extended Data Figure 3 | Elemental images for sample SQR-006. The correct orientation of the images can be seen in either Figure 2 or Extended Data Figure 2. The spatial resolution of the images is 0.186 mm per pixel. The shape of the sample in the elemental images looks different from the thin section sample (see Extended Data Figure 2) because the latter got broken during shipment from Brazil to the laboratory in Japan. Cold and warm colours in the elemental images represent lowest and highest X-ray counts (Al: 0 – 12; Si: 0 – 64; S: 0 – 22; K: 0 – 68; Ca: 0 – 32; Ti: 0 – 427; Fe: 0 – 728; Cu: 0 – 23).

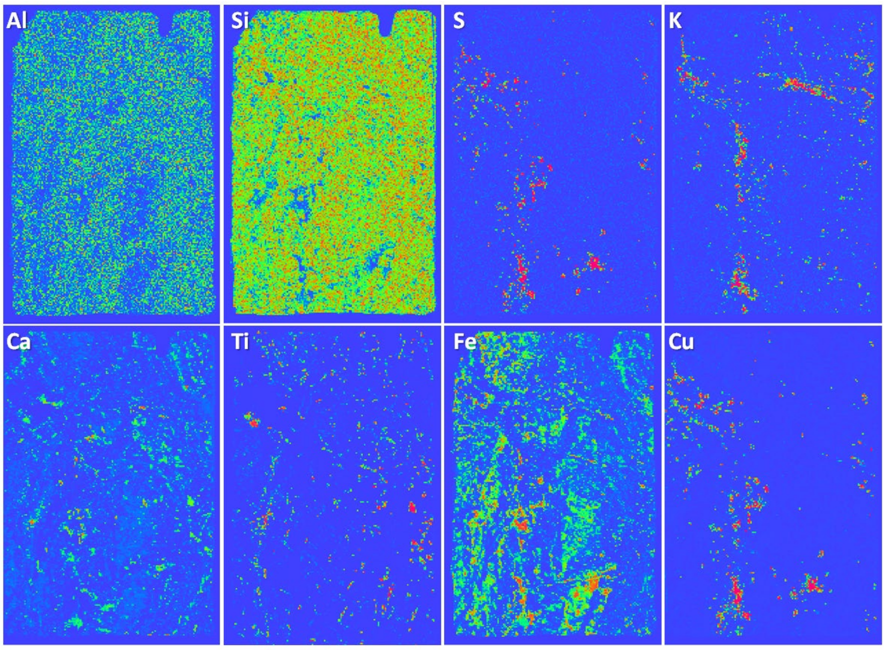

SQR-008

Extended Data Figure 4 | Elemental images for sample SQR-008. The correct orientation of the images can be seen in either Figure 2 or Extended Data Figure 2. The spatial resolution of the images is 0.190 mm per pixel. Cold and warm colours in the elemental images represent lowest and highest X-ray counts (Al: 0 – 12; Si: 0 – 41; S: 0 – 80; K: 0 – 76; Ca: 0 – 451; Ti: 0 – 485; Fe: 0 – 1042; Cu: 0 – 631).

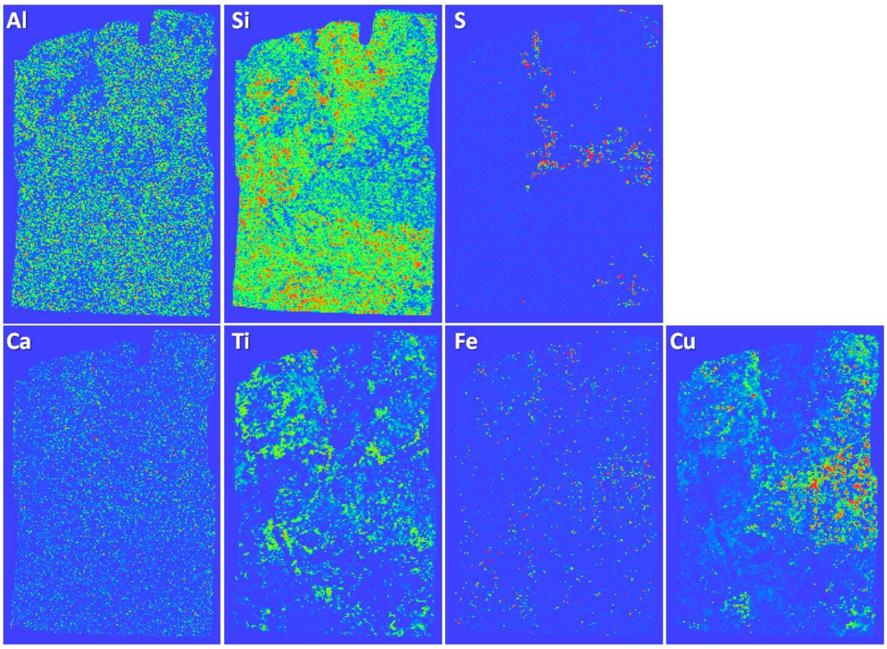

SQR-010

Extended Data Figure 5 | Elemental images for sample SQR-010. The correct orientation of the images can be seen in either Figure 2 or Extended Data Figure 2. The spatial resolution of the images is 0.184 mm per pixel. Image for K is absent because potassic alteration is not present in this sample. Cold and warm colours in the elemental images represent lowest and highest X-ray counts (Al: 0 – 11; Si: 0 – 67; S: 0 – 85; Ca: 0 – 11; Ti: 0 – 296; Fe: 0 – 360; Cu: 0 – 1895).

18

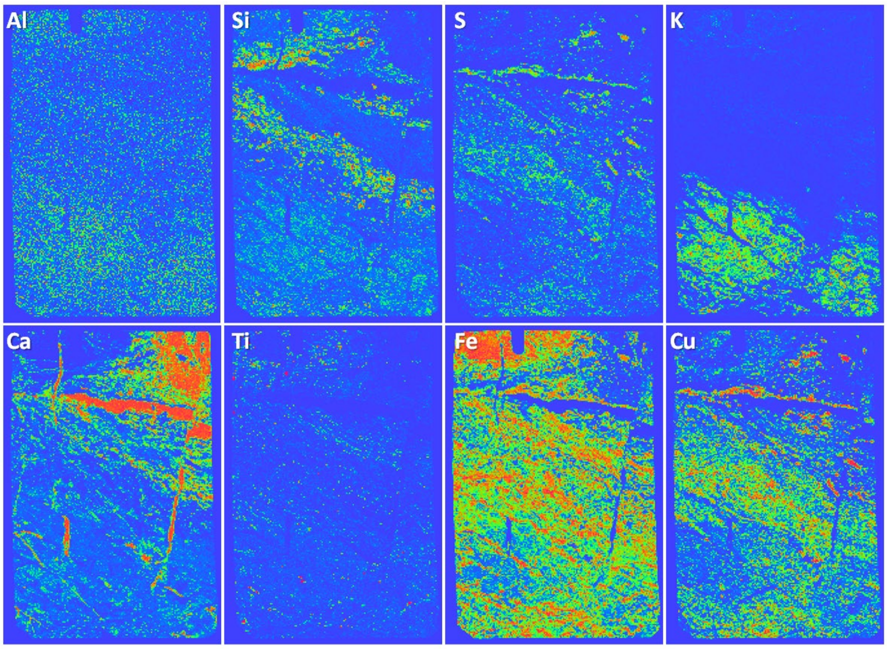

SQR-011

Extended Data Figure 6 | Elemental images for sample SQR-011. The correct orientation of the images can be seen in either Figure 2 or Extended Data Figure 2. The spatial resolution of the images is 0.176 mm per pixel. Cold and warm colours in the elemental images represent lowest and highest X-ray counts (Al: 0 – 10; Si: 0 – 62; S: 0 – 97; K: 0 – 83; Ca: 0 – 697; Ti: 0 – 642; Fe: 0 – 1127; Cu: 0 – 628).

19

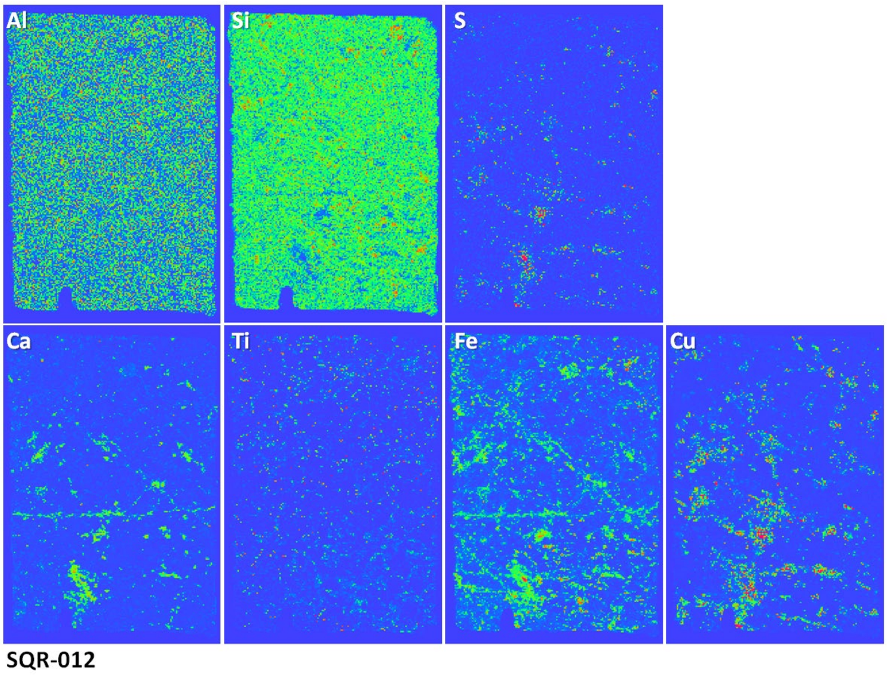

Extended Data Figure 7 | Elemental images for sample SQR-012. The correct orientation of the images can be seen in either Figure 2 or Extended Data Figure 2. The spatial resolution of the images is 0.184 mm per pixel. Image for K is absent because potassic alteration is not present in this sample. Cold and warm colours in the elemental images represent lowest and highest X-ray counts (Al: 0 – 11; Si: 0 – 62; S: 0 – 125; Ca: 0 – 274; Ti: 0 – 215; Fe: 0 – 946; Cu: 0 – 526).

20

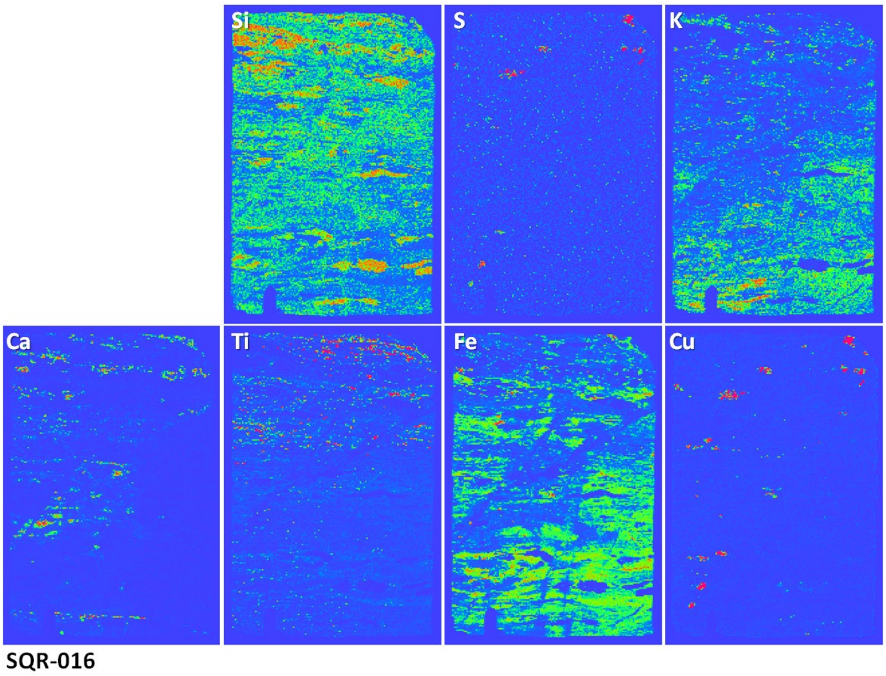

Extended Data Figure 8 | Elemental images for sample SQR-016. The correct orientation of the images can be seen in either Figure 2 or Extended Data Figure 2. The spatial resolution of the images is 0.184 mm per pixel. Image for Al is absent because this element had very low sensitivity in this sample. Cold and warm colours in the elemental images represent lowest and highest X-ray counts (Si: 0 – 67; S: 0 – 71; K: 0 – 75; Ca: 0 – 523; Ti: 0 – 541; Fe: 0 – 1778; Cu: 0 – 1003).

21

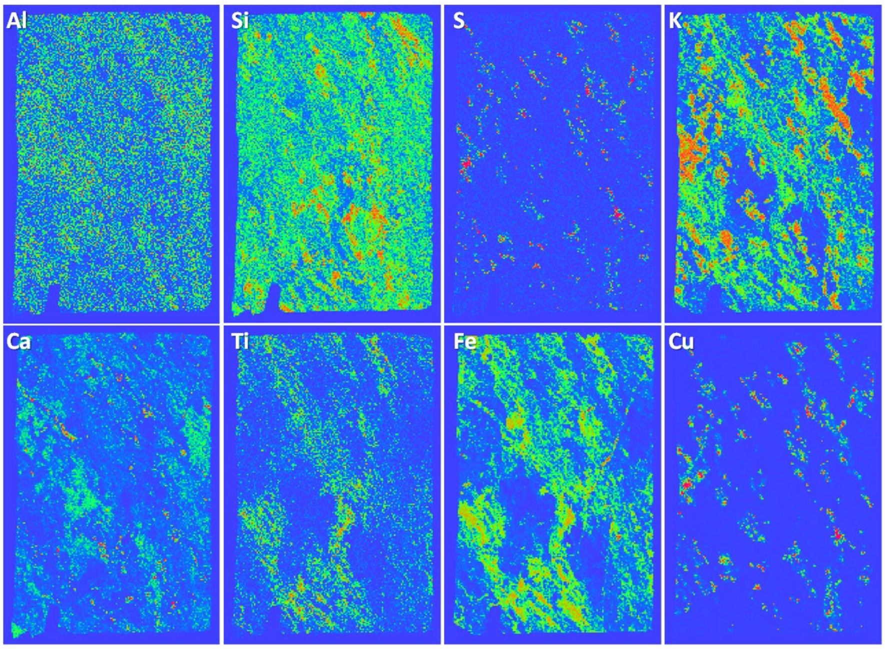

SQR-018

Extended Data Figure 9 | Elemental images for sample SQR-018. The correct orientation of the images can be seen in either Figure 2 or Extended Data Figure 2. The spatial resolution of the images is 0.184 mm per pixel. Cold and warm colours in the elemental images represent lowest and highest X-ray counts (Al: 0 – 11; Si: 0 – 60; S: 0 – 58; K: 0 – 79; Ca: 0 – 239; Ti: 0 – 19; Fe: 0 – 1219; Cu: 0 – 580).

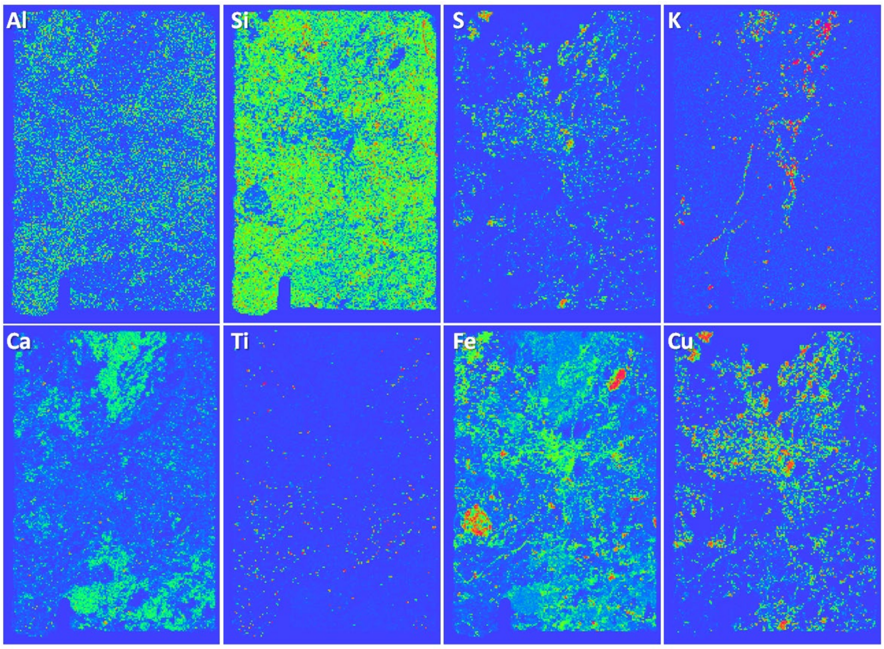

SQR-021

Extended Data Figure 10 | Elemental images for sample SQR-021. The correct orientation of the images can be seen in either Figure 2 or Extended Data Figure 2. The spatial resolution of the images is 0.184 mm per pixel. Cold and warm colours in the elemental images represent lowest and highest X-ray counts (Al: 0 – 11; Si: 0 – 49; S: 0 – 89; K: 0 – 53; Ca: 0 – 236; Ti: 0 – 215; Fe: 0 – 1840; Cu: 0 – 703).

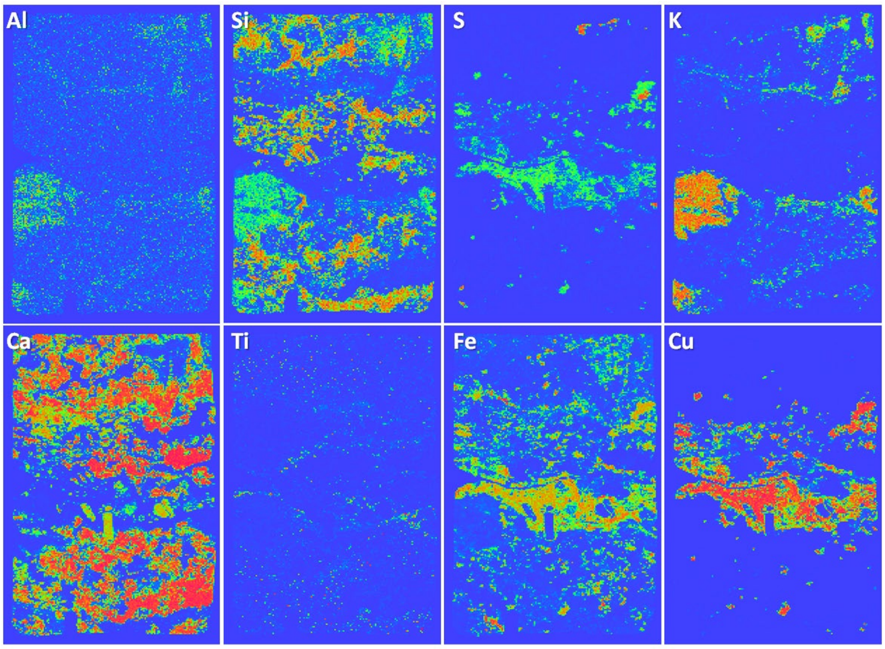

SSG-002

Extended Data Figure 11 | Elemental images for sample SSG-002. The correct orientation of the images can be seen in either Figure 2 or Extended Data Figure 2. The spatial resolution of the images is 0.184 mm per pixel. Cold and warm colours in the elemental images represent lowest and highest X-ray counts (Al: 0 – 12; Si: 0 – 65; S: 0 – 152; K: 0 – 92; Ca: 0 – 592; Ti: 0 – 213; Fe: 0 – 1271; Cu: 0 – 671).

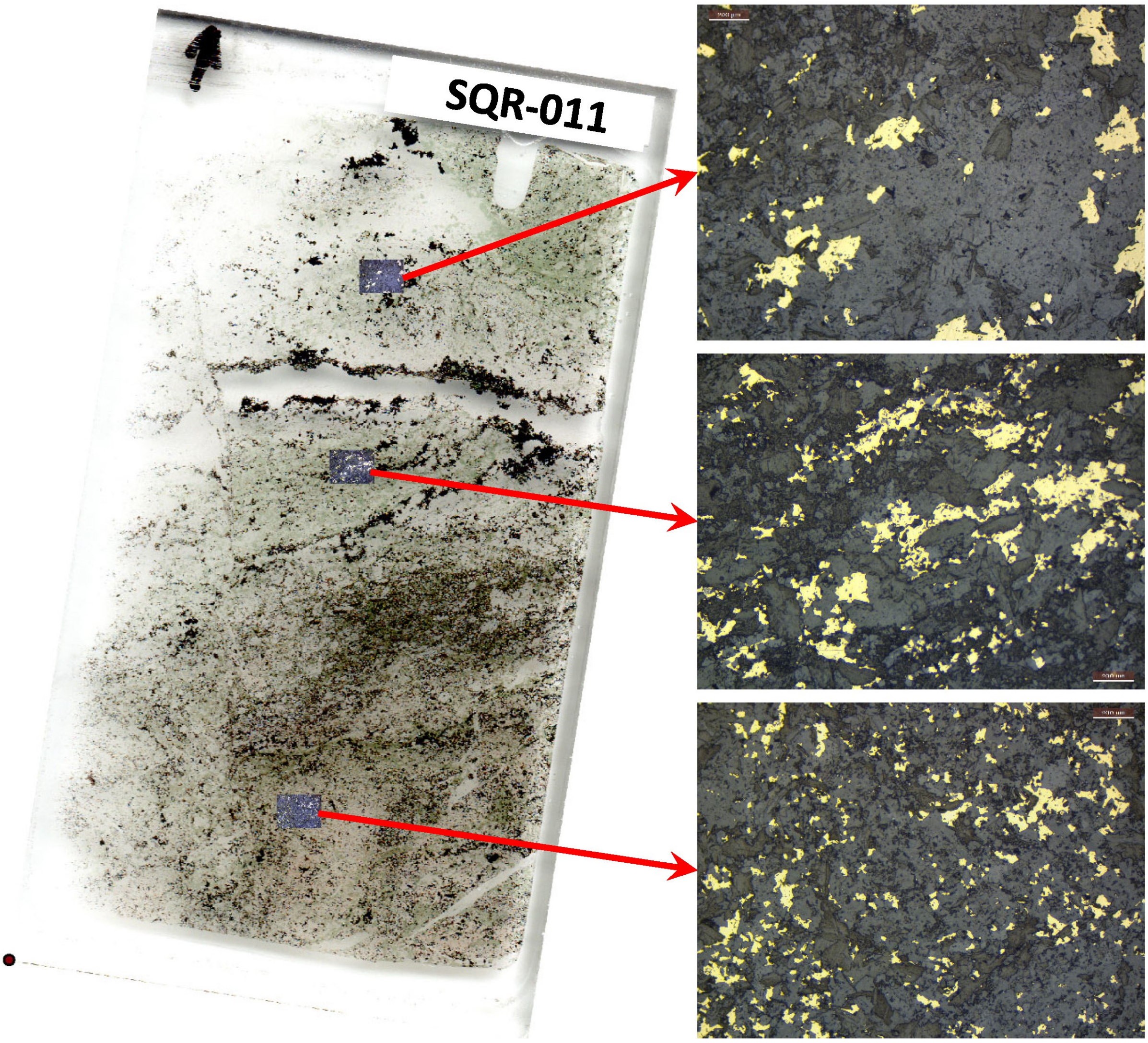

Extended Data Figure 12 | Predominant ore mineral in thin sections. An image of a representative thin section that is georeferenced in a geographic information system (GIS), and images of three photomicrographs obtained from this thin section under reflected light. Yellow minerals are chalcopyrite, which is the chief ore mineral in the studied deposit.

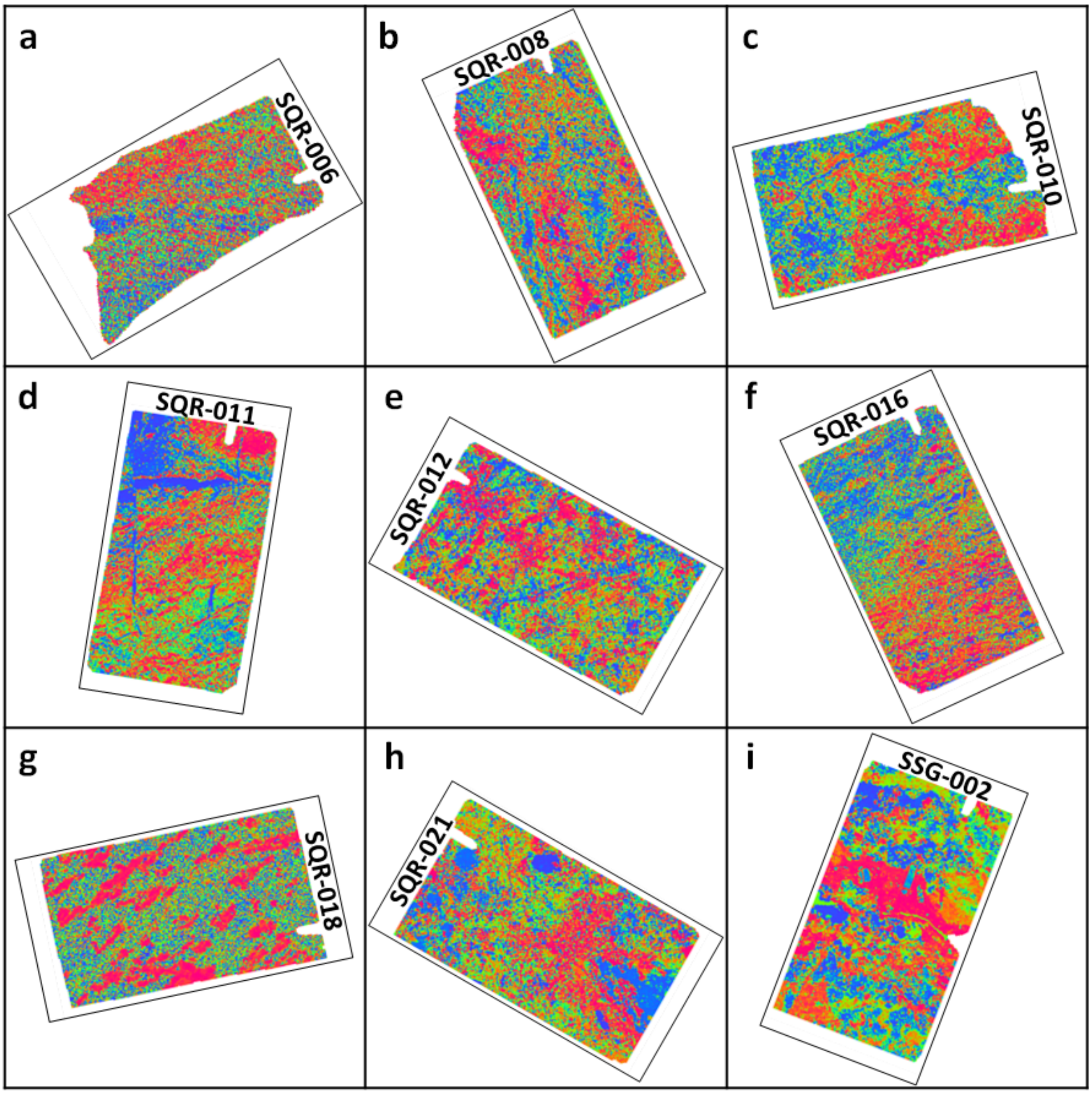

Extended Data Figure 13 | Spatial distributions of PC scores depicting geochemical signatures of mineralization in each thin section specimen. Cold and warm colours in the elemental images represent lowest and highest PC scores ("mineralization intensities"), but the ranges of PC scores are different in individual images. The thin sections used in this study are shown in their correct orientations with respect to the geological map (i.e., the North-South orientation is parallel to the long-side of this page).

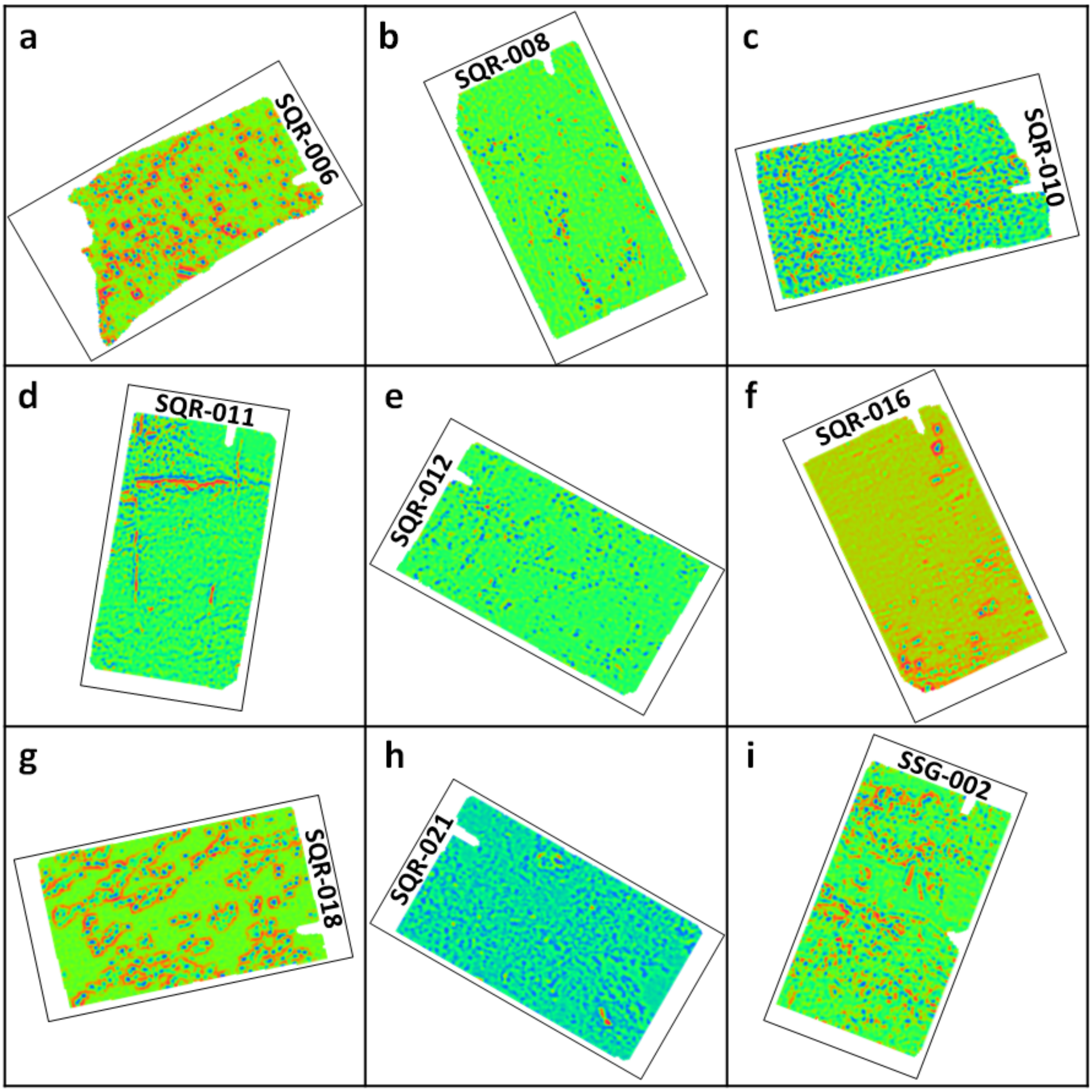

Extended Data Figure 14 | Spatial distributions of singularity indices derived from images of PC scores depicting geochemical signatures of mineralization in each thin section specimen (Extended Data Figure 12). Cold and warm colours in each of the images represent lowest and highest singularity indices, but the ranges of singularity indices are different in individual images. The thin sections used in this study are shown in their correct orientations with respect to the geological map (i.e., the North-South orientation is parallel to the long-side of this page).

35

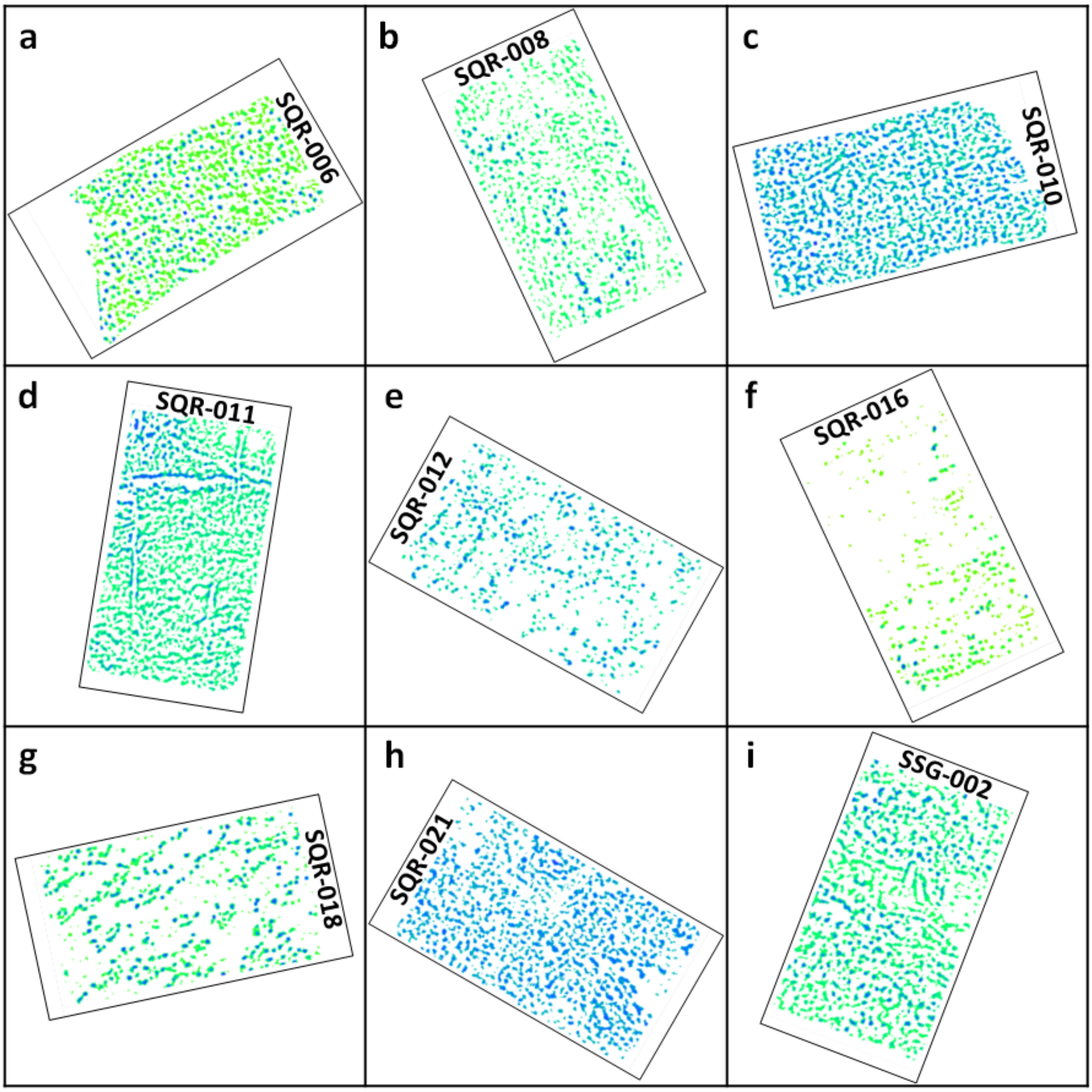

Extended Data Figure 15 | Spatial distributions of geochemical anomalies (i.e., pixels with singularity indices < 2) in images of PC scores depicting geochemical signatures of mineralization in each thin section specimen (Extended Data Figure 12). Blue and green pixels in each of the images have lowest and highest singularity indices < 2, but the ranges of singularity indices are different in individual images. The thin sections used in this study are shown in their correct orientations with respect to the geological map (i.e., the North-South orientation is parallel to the long-side of this page).

36

37

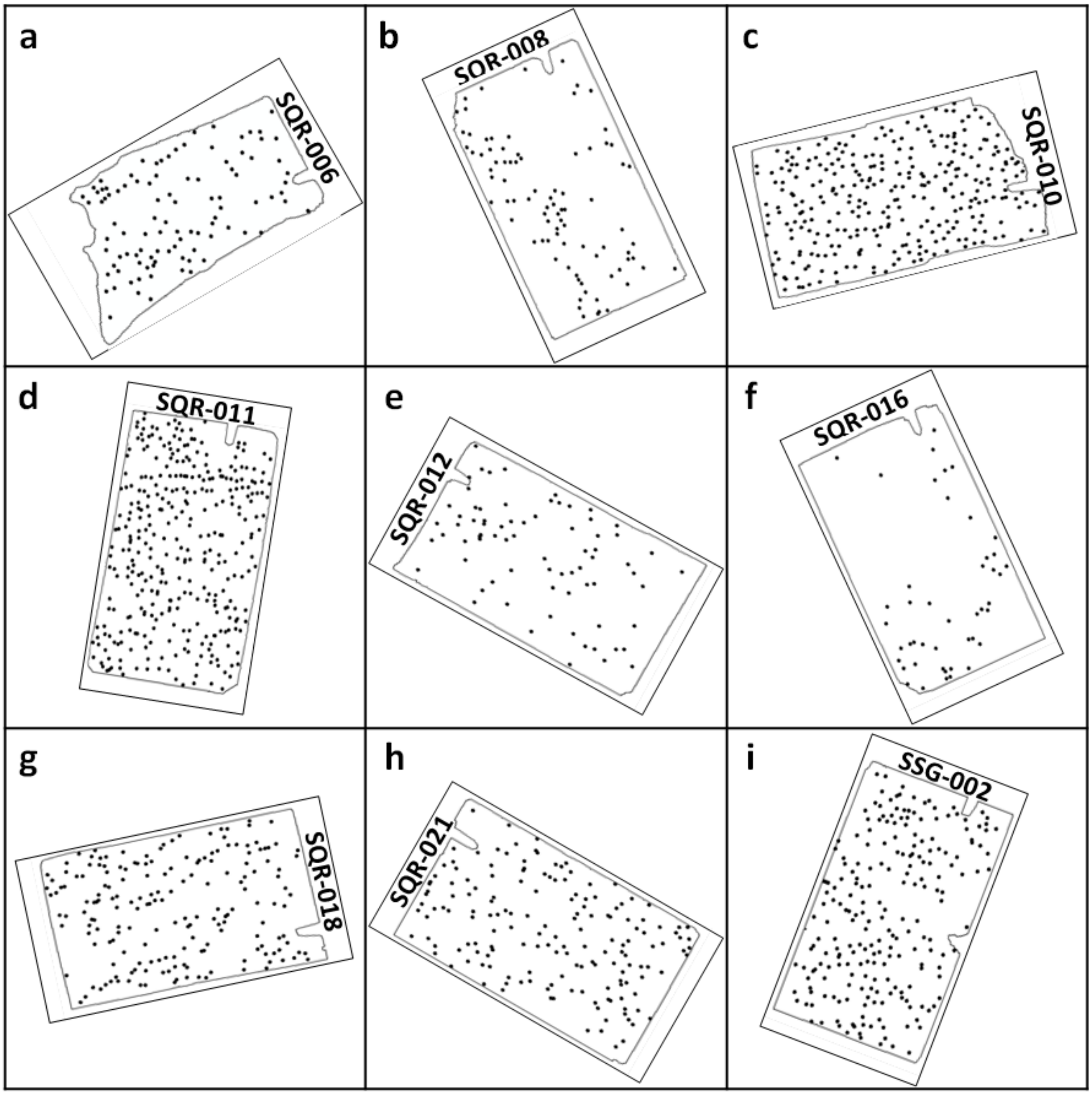

Extended Data Figure 16 | 'Geochemical anomaly centres' or loci of mineralization in each thin section specimen. Black dots are pixels with the least singularity indices in a 3 × 3 neighbourhood of pixels in every image. The thin sections used in this study are shown in their correct orientations with respect to the geological map (i.e., the North-South orientation is parallel to the long-side of this page).

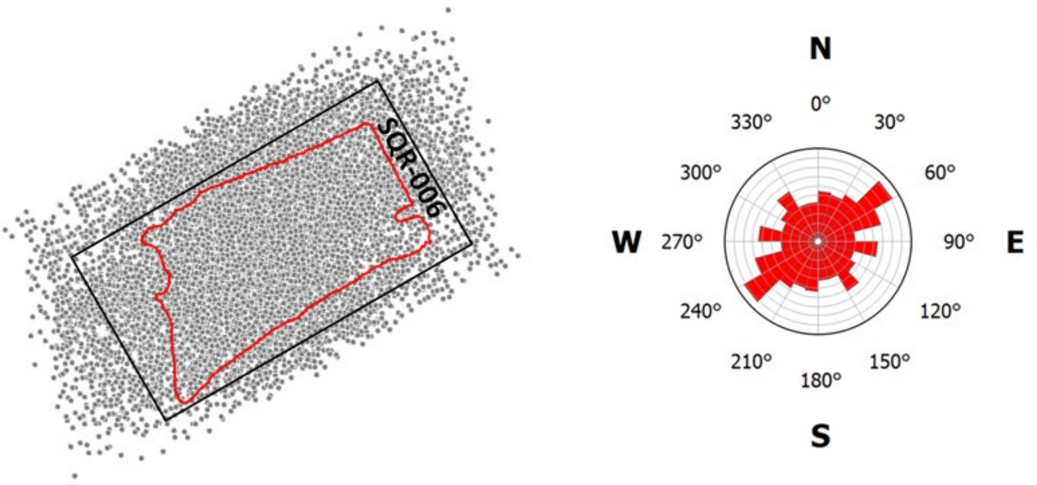

Extended Data Figure 17 | Fry plots and trends of 'geochemical anomaly centres' or loci of mineralization in thin sample SQR-006. Fry points are shown in grey dots. The rose diagram shows trends of pairs of Fry points that are <4.5 mm apart The thin section is shown in their correct orientations with respect to the geological map (i.e., the North-South orientation is parallel to the long-side of this page).

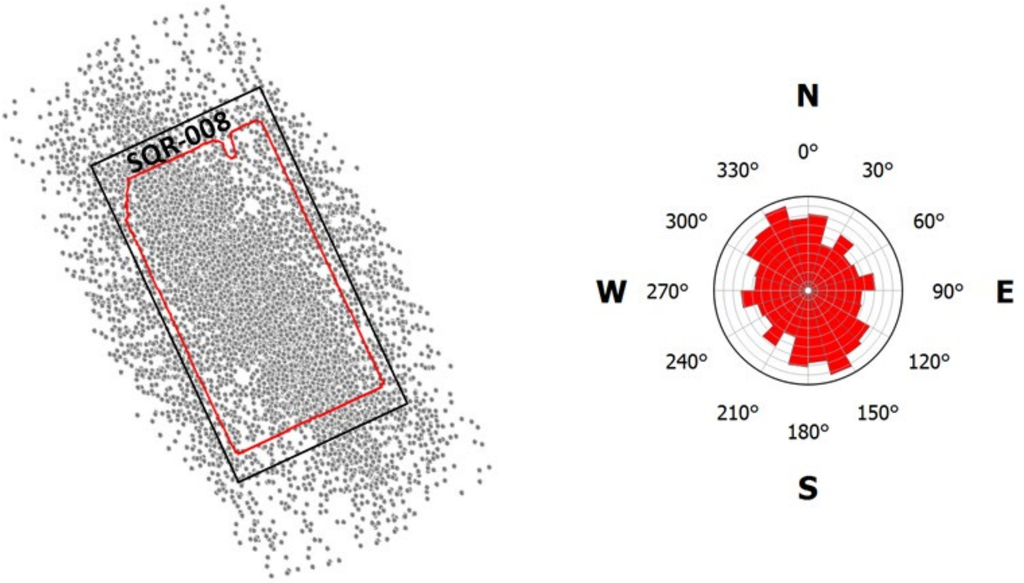

Extended Data Figure 18 | Fry plots and trends of 'geochemical anomaly centres' or loci of mineralization in thin sample SQR-008. Fry points are shown in grey dots. The rose diagram shows trends of pairs of Fry points that are <7 mm apart The thin section is shown in their correct orientations with respect to the geological map (i.e., the North-South orientation is parallel to the long-side of this page).

45

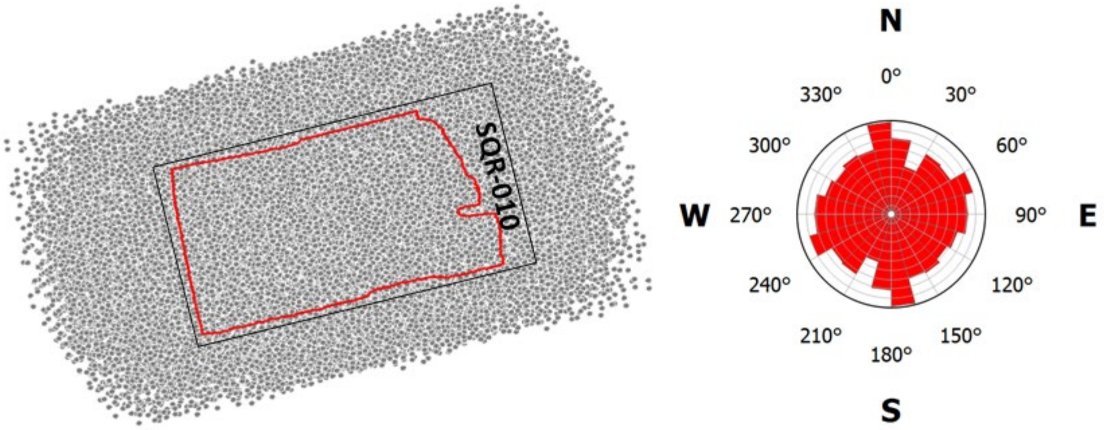

Extended Data Figure 19 | Fry plots and trends of 'geochemical anomaly centres' or loci of mineralization in thin sample SQR-010. Fry points are shown in grey dots. The rose diagram shows trends of pairs of Fry points that are <2.5 mm apart The thin section is shown in their correct orientations with respect to the geological map (i.e., the North-South orientation is parallel to the long-side of this page).

46

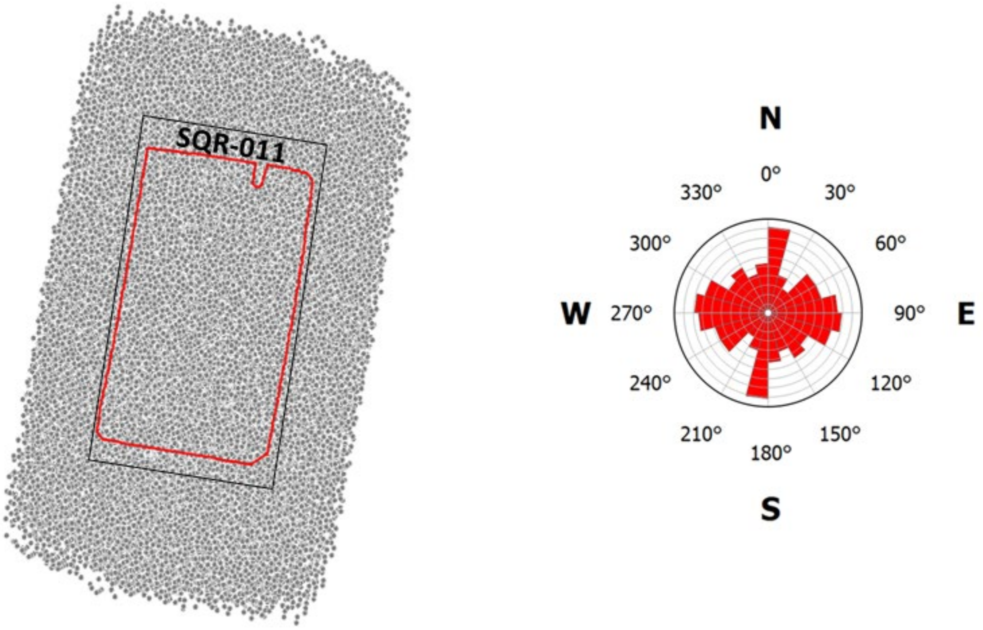

Extended Data Figure 20 | Fry plots and trends of 'geochemical anomaly centres' or loci of mineralization in thin sample SQR-011. Fry points are shown in grey dots. The rose diagram shows trends of pairs of Fry points that are <2 mm apart The thin section is shown in their correct orientations with respect to the geological map (i.e., the North-South orientation is parallel to the long-side of this page).

47

48

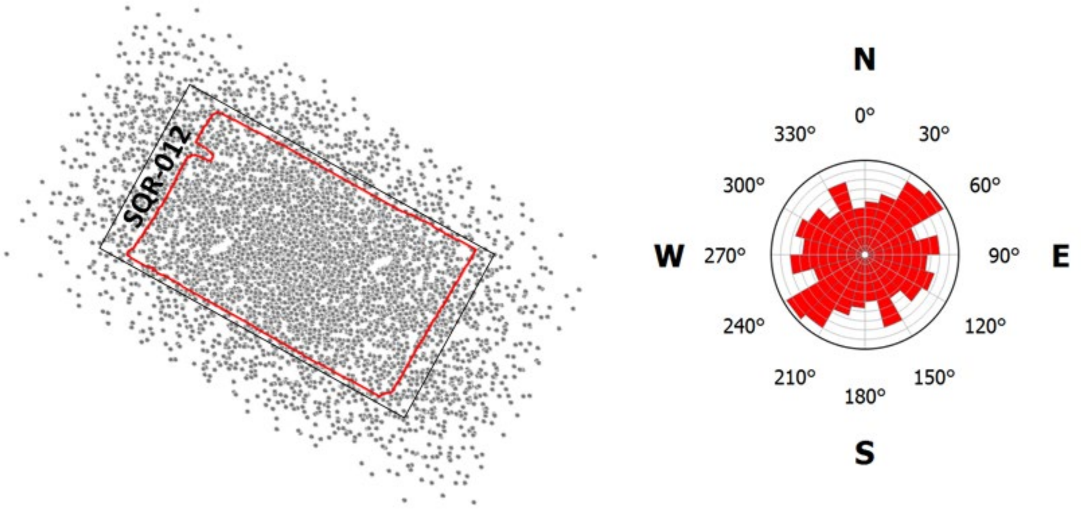

Extended Data Figure 21 | Fry plots and trends of 'geochemical anomaly centres' or loci of mineralization in thin sample SQR-012. Fry points are shown in grey dots. The rose diagram shows trends of pairs of Fry points that are <5.5 mm apart The thin section is shown in their correct orientations with respect to the geological map (i.e., the North-South orientation is parallel to the long-side of this page).

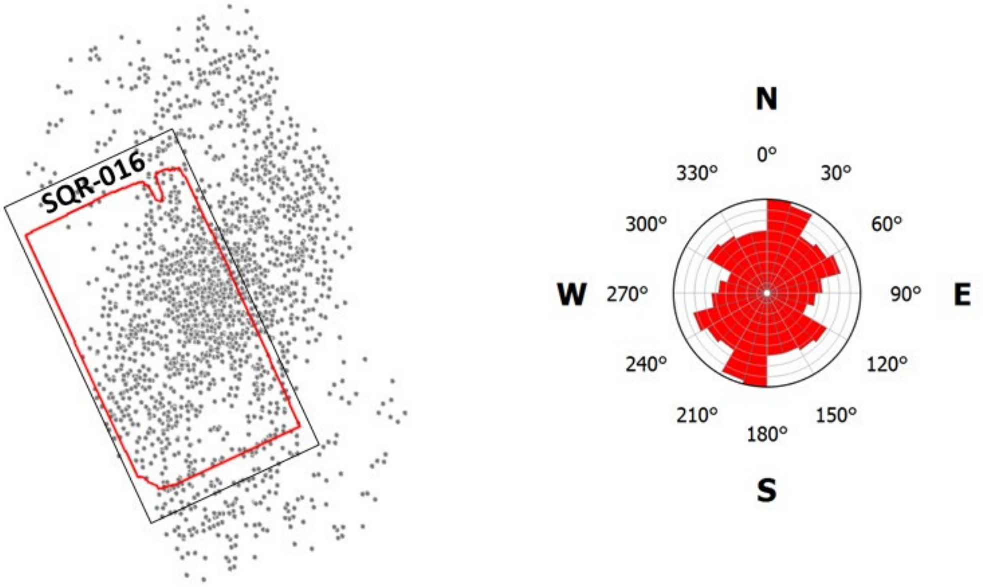

Extended Data Figure 22 | Fry plots and trends of 'geochemical anomaly centres' or loci of mineralization in thin sample SQR-016. Fry points are shown in grey dots. The rose diagram shows trends of pairs of Fry points that are <8.2 mm apart The thin section is shown in their correct orientations with respect to the geological map (i.e., the North-South orientation is parallel to the long-side of this page).

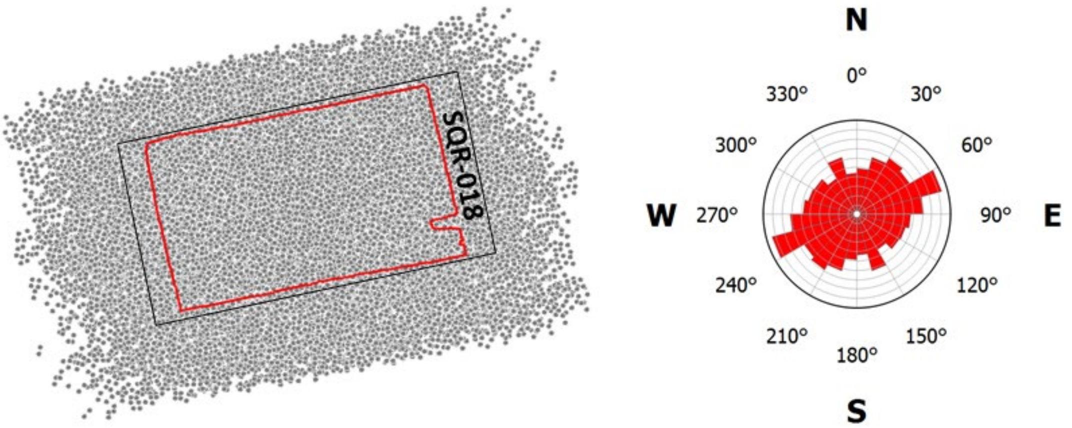

Extended Data Figure 23 | Fry plots and trends of 'geochemical anomaly centres' or loci of mineralization in thin sample SQR-018. Fry points are shown in grey dots. The rose diagram shows trends of pairs of Fry points that are <3.5 mm apart The thin section is shown in their correct orientations with respect to the geological map (i.e., the North-South orientation is parallel to the long-side of this page).

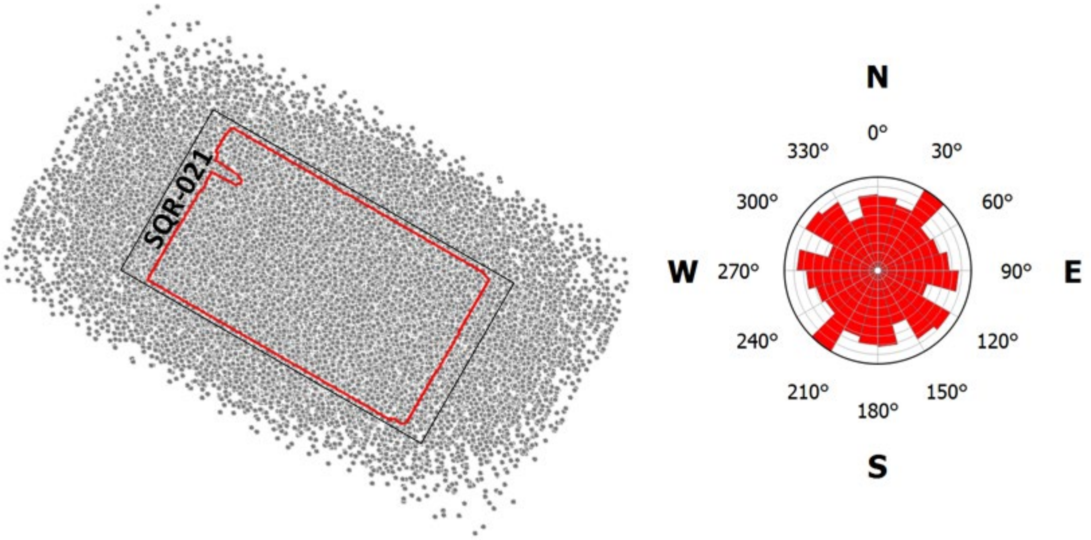

Extended Data Figure 24 | Fry plots and trends of 'geochemical anomaly centres' or loci of mineralization in thin sample SQR-021. Fry points are shown in grey dots. The rose diagram shows trends of pairs of Fry points that are <4 mm apart The thin section is shown in their correct orientations with respect to the geological map (i.e., the North-South orientation is parallel to the long-side of this page).

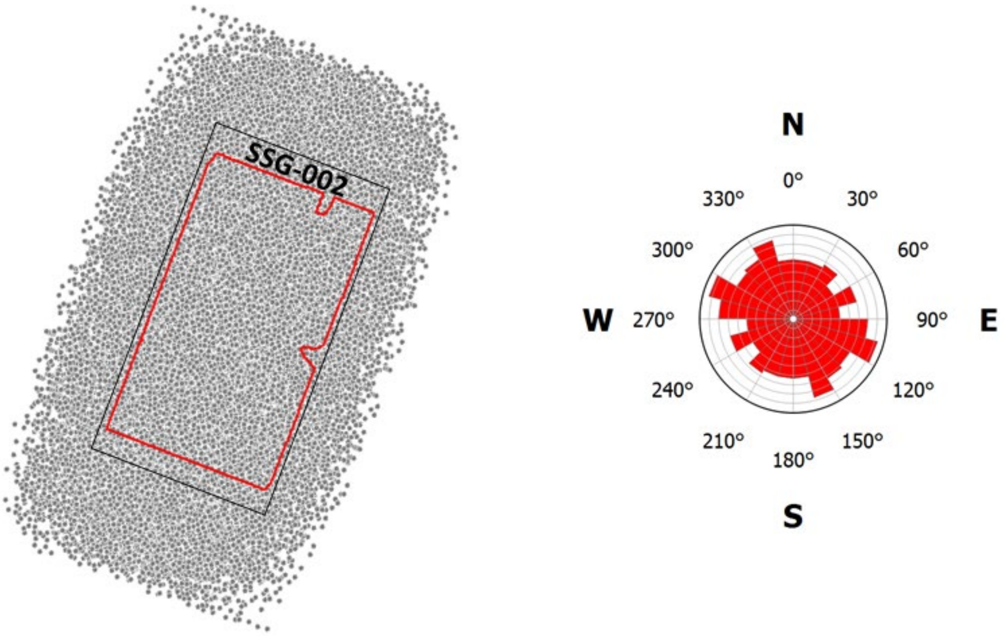

Extended Data Figure 25 | Fry plots and trends of 'geochemical anomaly centres' or loci of mineralization in thin sample SSG-002. Fry points are shown in grey dots. The rose diagram shows trends of pairs of Fry points that are <2 mm apart The thin section is shown in their correct orientations with respect to the geological map (i.e., the North-South orientation is parallel to the long-side of this page).

Extended Data Table 1 | PC analysis of SXAM elemental images for sample SQR-006 (Extended Data Figure 3). The bold text represents PC that depicts the geochemical signature of mineralization.

	Al	Si	S	K	Ca	Ti	Fe	Cu	Variance (%)	Cumulative variance (%)
**PC1**	**0.003**	**0.047**	**-0.003**	**-0.002**	**-0.002**	**-0.313**	**-0.949**	**0.000**	**64.11**	**64.11**
PC2	0.002	0.091	-0.010	-0.009	-0.003	-0.944	0.316	-0.005	26.49	90.60
PC3	0.044	-0.993	-0.003	0.045	0.011	-0.101	-0.016	0.016	7.63	98.23
PC4	0.102	-0.041	-0.002	-0.994	0.011	0.005	-0.001	-0.003	0.80	99.03
PC5	0.993	0.049	0.003	0.100	0.033	0.007	0.003	0.017	0.38	99.41
PC6	-0.017	0.015	-0.002	-0.006	0.002	-0.003	0.002	1.000	0.34	99.75
PC7	0.034	-0.010	-0.001	-0.007	-0.999	0.003	0.001	0.003	0.17	99.92
PC8	0.003	0.003	-1.000	0.002	0.001	0.010	-0.000	-0.002	0.08	100.00

Extended Data Table 2 | PC analysis of SXAM elemental images for sample SQR-008 (Extended Data Figure 4). The bold text represents PC that depicts the geochemical signature of mineralization.

	Al	Si	S	K	Ca	Ti	Fe	Cu	Variance (%)	Cumulative variance (%)
PC1	-0.002	-0.020	0.009	0.001	0.071	-0.000	0.991	0.110	84.86	84.86
**PC2**	**-0.002**	**-0.013**	**-0.062**	**-0.019**	**0.502**	**0.236**	**0.056**	**-0.828**	**7.33**	**92.19**
PC3	-0.004	-0.039	0.043	0.012	0.622	0.566	-0.104	0.528	5.22	97.41
PC4	-0.002	0.013	-0.012	-0.011	-0.596	0.790	0.058	-0.131	2.40	99.81
PC5	-0.015	-0.123	0.034	-0.991	-0.000	-0.009	-0.004	0.019	0.09	99.90
PC6	-0.018	-0.991	0.011	0.124	-0.040	-0.014	-0.015	-0.018	0.08	99.98
PC7	0.053	-0.018	-0.995	-0.031	0.003	0.000	0.000	0.077	0.01	99.99
PC8	-0.998	0.019	-0.054	0.011	-0.001	-0.004	-0.002	0.004	0.01	100.00

Extended Data Table 3 | PC analysis of SXAM elemental images for sample SQR-010 (Extended Data Figure 5). The bold text represents PC that depicts the geochemical signature of mineralization.

	Al	Si	S	Ca	Ti	Fe	Cu	Variance (%)	Cumulative variance (%)
**PC1**	**-0.001**	**-0.020**	**0.003**	**0.000**	**0.048**	**0.005**	**0.999**	**98.26**	**98.26**
PC2	0.002	-0.079	-0.010	0.002	0.996	0.012	-0.050	1.55	99.81
PC3	0.001	0.102	-0.001	-0.001	0.020	-0.995	0.006	0.09	99.90
PC4	-0.049	0.986	-0.088	-0.005	0.077	0.103	0.015	0.08	99.98
PC5	0.043	-0.084	-0.995	-0.007	-0.016	-0.008	0.002	0.02	100.00
PC6	-0.998	-0.052	-0.039	0.012	-0.002	-0.006	-0.002	0.00	100.00
PC7	0.012	0.005	-0.007	1.000	-0.002	-0.001	-0.000	0.00	100.00

Extended Data Table 4 | PC analysis of SXAM elemental images for sample SQR-011 (Extended Data Figure 6). The bold text represents PC that depicts the geochemical signature of mineralization.

	Al	Si	S	K	Ca	Ti	Fe	Cu	Variance (%)	Cumulative variance (%)
**PC1**	**0.001**	**-0.001**	**0.014**	**-0.006**	**-0.422**	**0.004**	**0.886**	**0.192**	**76.68**	**76.68**
PC2	0.001	-0.013	-0.067	-0.013	0.306	-0.002	0.339	-0.887	14.03	90.71
PC3	-0.003	-0.063	0.042	-0.055	0.850	-0.016	0.314	0.412	8.98	99.69
PC4	-0.002	0.065	-0.002	-0.046	-0.014	-0.997	-0.002	-0.003	0.15	99.84
PC5	0.040	-0.223	-0.006	0.971	0.034	-0.060	0.021	0.009	0.10	99.94
PC6	-0.013	-0.732	0.654	-0.163	-0.057	-0.040	-0.025	-0.066	0.03	99.97
PC7	0.003	-0.637	-0.752	-0.151	-0.038	-0.033	-0.018	0.048	0.02	99.99
PC8	-0.999	-0.002	-0.011	0.040	-0.000	-0.000	0.001	-0.001	0.01	100.00

Extended Data Table 5 | PC analysis of SXAM elemental images for sample SQR-012 (Extended Data Figure 7). The bold text represents PC that depicts the geochemical signature of mineralization.

	Al	Si	S	Ca	Ti	Fe	Cu	Variance (%)	Cumulative variance (%)
**PC1**	**-0.002**	**-0.019**	**0.009**	**0.111**	**0.034**	**0.097**	**0.988**	**89.21**	**89.21**
PC2	-0.001	0.000	0.052	-0.001	-0.059	0.992	-0.095	7.42	96.63
PC3	-0.002	0.043	0.007	-0.993	-0.008	0.009	0.112	2.37	99.00
PC4	-0.002	-0.054	-0.007	-0.014	0.996	0.056	-0.039	0.71	99.71
PC5	-0.014	0.997	-0.024	0.044	0.055	0.005	0.012	0.24	99.95
PC6	-0.030	0.023	0.998	0.007	0.011	-0.052	-0.004	0.03	99.98
PC7	0.999	0.015	0.029	-0.001	0.003	-0.000	0.002	0.02	100.00

Extended Data Table 6 | PC analysis of SXAM elemental images for sample SQR-016 (Extended Data Figure 8). The bold text represents PC that depicts the geochemical signature of mineralization.

	Si	S	K	Ca	Ti	Fe	Cu	Variance (%)	Cumulative variance (%)
PC1	-0.023	0.000	0.013	-0.019	0.020	0.999	0.000	95.68	95.68
PC2	0.050	-0.007	0.026	-0.996	0.000	-0.018	-0.067	2.36	98.04
PC3	-0.022	-0.026	-0.042	0.028	0.892	-0.017	-0.447	0.92	98.96
**PC4**	**0.019**	**-0.052**	**0.045**	**0.062**	**-0.447**	**0.010**	**-0.890**	**0.86**	**99.82**
PC5	-0.283	-0.001	0.958	0.010	0.047	-0.020	0.020	0.12	99.94
PC6	-0.957	0.005	-0.280	-0.054	-0.044	-0.019	-0.017	0.06	100.00
PC7	0.006	0.998	0.004	-0.002	-0.000	-0.000	-0.058	0.00	100.00

Extended Data Table 7 | PC analysis of SXAM elemental images for sample SQR-018 (Extended Data Figure 9). The bold text represents PC that depicts the geochemical signature of mineralization.

	Al	Si	S	K	Ca	Ti	Fe	Cu	Variance (%)	Cumulative variance (%)
PC1	0.002	0.016	-0.001	-0.023	0.033	-0.009	-0.999	0.000	94.14	94.14
**PC2**	**-0.001**	**0.005**	**-0.060**	**-0.114**	**0.046**	**0.008**	**0.004**	**-0.991**	**4.58**	**98.72**
PC3	-0.003	-0.090	0.027	-0.595	0.791	-0.013	0.039	0.103	0.73	99.45
PC4	0.032	-0.292	-0.018	0.773	0.558	0.018	-0.004	-0.063	0.42	99.87
PC5	0.036	-0.950	0.020	-0.183	-0.246	0.028	-0.019	0.003	0.10	99.97
PC6	-0.039	-0.032	0.014	0.017	-0.007	-0.998	0.008	-0.011	0.01	99.98
PC7	-0.998	-0.042	0.020	0.020	0.007	0.041	-0.003	-0.002	0.01	99.99
PC8	0.021	0.018	0.997	0.027	-0.004	0.013	-0.002	-0.064	0.01	100.00

Extended Data Table 8 | PC analysis of SXAM elemental images for sample SSG-002 (Extended Data Figure 10). The bold text represents PC that depicts the geochemical signature of mineralization.

	Al	Si	S	K	Ca	Ti	Fe	Cu	Variance (%)	Cumulative variance (%)
PC1	-0.002	-0.015	0.016	0.001	0.016	0.001	0.980	0.195	84.04	84.04
**PC2**	**0.000**	**0.010**	**-0.074**	**-0.003**	**0.082**	**0.007**	**0.194**	**-0.975**	**15.07**	**99.11**
PC3	-0.018	-0.032	0.013	0.002	0.990	0.107	-0.033	0.077	0.70	99.81
PC4	0.007	-0.031	0.005	-0.007	-0.108	0.994	0.001	-0.003	0.11	99.92
PC5	-0.002	0.952	-0.297	0.047	0.028	0.034	0.012	0.038	0.03	99.95
PC6	-0.021	0.282	0.817	-0.499	0.004	0.002	0.002	-0.057	0.02	99.97
PC7	-0.012	-0.111	-0.488	-0.865	0.002	-0.007	-0.001	0.039	0.02	99.99
PC8	-0.999	-0.006	-0.011	0.021	-0.019	0.005	-0.001	-0.001	0.01	100.00

Extended Data Table 9 | PC analysis of SXAM elemental images for sample SQR-021 (Extended Data Figure 11). The bold text represents PC that depicts the geochemical signature of mineralization.

	Al	Si	S	K	Ca	Ti	Fe	Cu	Variance (%)	Cumulative variance (%)
**PC1**	**0.000**	**-0.004**	**0.041**	**0.001**	**-0.429**	**0.004**	**0.775**	**0.462**	**69.87**	**69.87**
PC2	0.002	0.075	-0.017	0.031	-0.900	0.003	-0.386	-0.187	21.80	91.67
PC3	-0.002	-0.005	0.077	-0.020	0.032	-0.015	-0.499	0.862	8.02	99.69
PC4	-0.038	0.292	-0.007	-0.955	-0.008	0.020	0.007	-0.015	0.17	99.86
PC5	0.002	0.487	-0.839	0.157	0.038	0.158	0.013	0.090	0.06	99.92
PC6	0.000	-0.110	0.119	-0.013	-0.003	0.987	-0.013	-0.002	0.05	99.97
PC7	0.010	0.812	0.524	0.245	0.061	0.031	0.024	-0.024	0.02	99.99
PC8	-0.999	-0.002	0.004	0.040	-0.000	0.000	0.001	-0.002	0.01	100.00
